# Berberine pharmacological properties and therapeutic potential across cancer, digestive, metabolic, cardiovascular, and neurological diseases: an update review

**DOI:** 10.17179/excli2025-8771

**Published:** 2025-09-08

**Authors:** Haoxuan Cheng, Xinyu Li, Yanqi Wang, Wanqing Deng, Guangyong Sun, Dong Zhang, Jianyu Hao, Xinjuan Liu

**Affiliations:** 1 Department of Gastroenterology, Beijing Chao-Yang Hospital, Capital Medical University, Beijing, China; 2 Department of Laboratory Medicine, Beijing Chao-Yang Hospital, Capital Medical University, Beijing, China

**Keywords:** berberine, coptis, pharmacological properties, clinical applications, cancer, gut microbiota

## Abstract

Berberine (BBR) is a plant-derived alkaloid that has been traditionally used in Chinese medicine to treat diarrhea. In recent years, accumulating evidence has highlighted its broad therapeutic potential across multiple organ systems. This review systematically examines the pharmacological mechanisms and therapeutic applications of BBR in cancer, as well as in digestive, metabolic, cardiovascular, and neurological diseases. The effects of BBR on endogenous factors-such as energy metabolism, immune responses, cellular homeostasis, and gene expression-are discussed, along with its regulation of cellular functions and inflammatory responses. In addition, we explore BBR's actions on exogenous factors, particularly the gut microbiota. The review also summarizes emerging molecular targets of BBR and addresses current clinical applications, as well as novel strategies to improve its low oral bioavailability. By integrating findings from basic, translational, and clinical research, this review provides a comprehensive overview of BBR's therapeutic potential and supports its integration into modern medical practice.

See also the graphical abstract(Fig. 1).

## Introduction

Berberine (BBR), a naturally occurring isoquinoline alkaloid of the protoberberine type derived from medicinal plants such as Coptis chinensis (Singh and Mahajan, 2013[161]), has been extensively utilized in traditional Chinese and Ayurvedic medicine for centuries, primarily treating gastrointestinal infections and related disorders (Gao et al., 2020[42]; Song et al., 2020[163]). Emerging preclinical and clinical evidence now reveals BBR's broad therapeutic potential across multiple organ systems (Harrison et al., 2021[52]; Chen et al., 2022[20]; Hu et al., 2024[61]). This multi-target activity suggests modulation of convergent molecular pathways in diverse pathologies. The present review systematically synthesizes current knowledge on BBR's pharmacological effects against system-related diseases, with critical analysis of its molecular mechanisms in cancer, digestive, metabolic, cardiovascular, and neurological disorders. Gaps in translational research and future directions are also discussed.

## Mechanistic Basis of BBR’s Systemic Effects: Metabolism, Immunity, Cellular Homeostasis, and Gene Regulation

BBR exerts its therapeutic effects through a complex network of endogenous regulatory mechanisms. Recent studies have increasingly focused on its roles in metabolic regulation, immune modulation, cellular homeostasis, and gene transcription, aiming to elucidate its multi-target pharmacological profile. These processes form the foundation of BBR's systemic effects and are key to understanding its broad therapeutic potential across a wide range of diseases.

### Metabolic regulation

**Figure 2:**
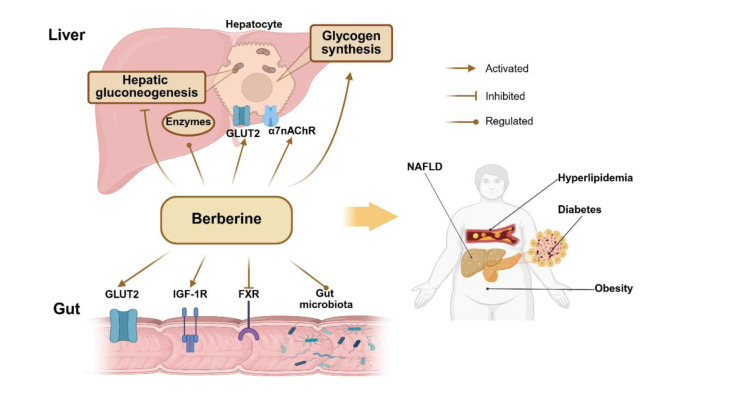
BBR regulates systemic metabolism by modulating the gut microbiota, key receptors and metabolic enzymes, gluconeogenesis, and glycogen synthesis. This figure was created by BioRender.com.

BBR alleviates various diseases by improving glucose and lipid metabolic disorders, primarily including diabetes, hyperlipidemia, and non-alcoholic fatty liver disease (NAFLD) (Figure 2(Fig. 2)).

#### Regulation of Glucose Metabolism

BBR regulates glucose metabolism through multiple mechanisms to maintain glycemic homeostasis.

First, BBR modulates glucose metabolism through the gut-liver axis. It inhibits phosphorylation of intestinal insulin-like growth factor 1 receptor (IGF-1R), reducing membrane localization of phospholipase C beta 2 (PLC-β2) and suppressing glucose transporter type 2 (GLUT2) translocation in intestinal epithelial cells, thereby decreasing glucose absorption (Zhang et al., 2021[257]). BBR also activates intestinal farnesoid X receptor (FXR) and downregulates hepatic glucose-6-phosphatase (G6Pase) and phosphoenolpyruvate carboxykinase (PEPCK) expression, reducing endogenous glucose production (Sun et al., 2021[169]). In addition, BBR inhibits mitochondrial respiration and pyruvate carboxylation, suppressing hepatic gluconeogenesis (Moreira et al., 2022[129]). Moreover, BBR upregulates hepatic glucokinase (GK) and phosphorylates protein phosphatase 2A (PP2A), promoting G6Pase production and phosphorylation of glycogen synthase kinase 3α (GSK-3α), which enhances hepatic glucose uptake and glycogen synthesis, increasing glycogen storage (Li et al., 2019[97]; Ren et al., 2020[151]; He et al., 2022[55]).

Second, BBR improves insulin resistance (IR). It activates PI3K/AKT and PPARγ signaling pathways, and increases GLUT2 expression (Chen et al., 2023[23]; Ma et al., 2024[122]). BBR also inhibits thioredoxin-interacting protein (TXNIP) to improve hepatic glucose uptake and reduces hypoxia-inducible factor 1-alpha (HIF-1α) expression to decrease ceramide accumulation in the liver of high-fat diet (HFD)-fed mice, thereby ameliorating IR (He et al., 2022[55]; Xia et al., 2022[217]). Additionally, by activating α7 nicotinic acetylcholine receptor (α7nAChR) and inhibiting the NF-κB pathway, BBR lowers proinflammatory cytokines such as TNF-α and IL-1β, alleviating inflammation-associated IR (Wang et al., 2022[182]).

BBR also affects glucose metabolism indirectly via modulation of gut microbiota (Sun et al., 2021[169]; Yang et al., 2022[240]) and lipid metabolism (Yang et al., 2024[241]), which will be discussed in subsequent sections.

#### Regulation of Lipid Metabolism

BBR improves obesity, hyperlipidemia, and NAFLD by regulating lipid metabolism.

BBR directly modulates hepatic lipid metabolism, primarily by regulating key enzymes involved in lipid metabolism. It binds to Aldo-keto reductase, reverses the abnormal expression of hepatic microsomal triglyceride transfer protein (MTTP), apolipoprotein B, and low-density lipoprotein receptor (LDLR), and inhibits fatty acid synthesis (Chen et al., 2021[18]; Yang et al., 2024[241]). BBR also activates the AMPK signaling pathway to regulate sirtuin 1 (SIRT1), a key enzyme in lipid metabolism, downregulates lipogenic genes, and upregulates fatty acid oxidation genes, alleviating oxidative stress (Yang et al., 2022[238]; Chen et al., 2024[12]). In addition, it mediates deacetylation of carnitine palmitoyl transferase 1A (CPT1A) at Lys675, reduces its ubiquitination and degradation, promotes β-oxidation of fatty acids, and significantly lowers hepatic triglyceride and cholesterol levels in NAFLD mice (Wang et al., 2022[189]). SIRT1 also promotes hepatocyte autophagy and activates fibroblast growth factor 21 (FGF21) to regulate hepatic lipid utilization (Sun et al., 2018[172]). Activation of the AMPK signaling pathway also inhibits stearoyl-CoA desaturase 1 (SCD1) expression, reducing hepatic triglyceride synthesis (Zhu et al., 2019[272]). BBR further inhibits mitochondrial complex I in the liver, decreases oxygen consumption rate and ATP production to suppress lipid synthesis, while promoting mitochondrial fusion, thereby alleviating obesity, hyperlipidemia, and NAFLD (Yu et al., 2021[252]). In addition, BBR combined with bicyclol inhibits hepatic stellate cell activation via the p62-nuclear factor erythroid 2 related factor 2 (Nrf2) -PPARα pathway, delaying liver fibrosis in the non-alcoholic steatohepatitis (NASH) model (Li et al., 2022[91]).

BBR also affects lipid metabolism by reshaping the gut microbiota and reducing intestinal lipid absorption. BBR increase beneficial bacteria such as Akkermansia and decrease pathogenic bacteria such as Lactobacillus, improving bile acid metabolism and indirectly modulating lipid metabolism (Yang et al., 2022[238][240]). BBR inhibits key enzymes involved in intestinal lipogenesis such as SCD1 and the receptor CD36 involved in fatty acid uptake, and enhances the “zippering” of lacteal tight junctions via the RhoA/ROCK signaling pathway, reducing fatty acid uptake and absorption, ultimately improving obesity and lipid metabolic disorders (Yu et al., 2021[252]; Wang et al., 2024[183]).

### Regulation of Immune Cells

**Figure 3:**
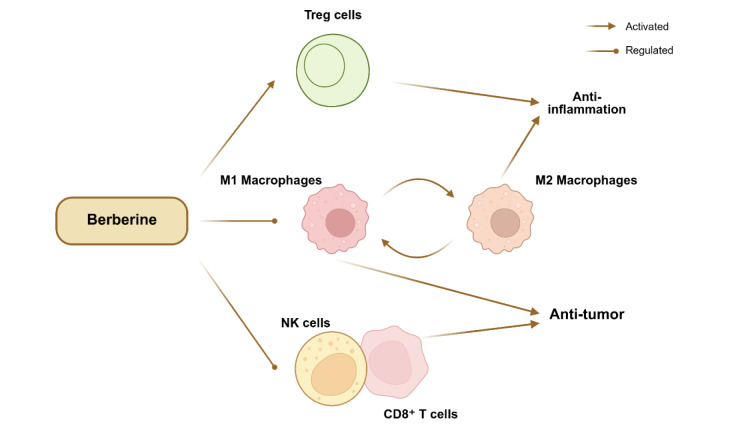
BBR exerts anti-tumor and anti-inflammatory effects by regulating macrophage polarization and modulating the functions of immune cells, including Treg cells, CD8⁺ T cells, and NK cells. This figure was created by BioRender.com.

BBR also modulates the functions of immune cells such as macrophages and T cells, exerting anti-inflammatory, immunoregulatory, and disease-alleviating effects in various conditions including tumors, digestive disorders, metabolic diseases, and neurological disorders (Figure 3(Fig. 3)).

#### Regulation of macrophage polarization

Macrophages, as key components of the immune system, activate other immune cells through antigen presentation, initiating and regulating immune responses, and exerting roles in immune defense and tissue repair. BBR modulates macrophage polarization and function, contributing to the treatment of various tumors and inflammatory diseases. In colitis-associated colorectal cancer (CRC), BBR inhibits macrophage IL-6/TNF-α expression, thereby blocking epidermal growth factor receptor (EGFR)-ERK signaling-induced inflammation (Li et al., 2017[87]). It also downregulates miR-155-5p to upregulate Suppressor of Cytokine Signaling 1 (SOCS1) expression, reducing M1 polarization and suppressing colitis-associated CRC progression (Ling et al., 2023[109]). In melanoma models, BBR promotes the conversion of M2 macrophages into pro-inflammatory M1 macrophages, restoring T cell anti-tumor activity (Shah et al., 2022[155]). During lung cancer progression, BBR inhibits Peptidyl Arginine Deiminase Type IV (PADI4), thereby reversing PADI4-mediated suppression of Interferon Regulatory Factor 5 (IRF5), enhancing M1 polarization and preventing lung tumorigenesis (Gu et al., 2022[46]). 

In addition to its regulatory effects on macrophages in tumor-related diseases, BBR also modulates macrophage polarization in inflammatory, metabolic and neurological disorders. In chronic atrophic gastritis (CAG) induced by Helicobacter pylori (H. pylori) and Lipopolysaccharide (LPS), BBR activates the IL-4-STAT6 pathway to inhibit M1 and promote M2 polarization, facilitating H. pylori clearance and disease remission (Yang et al., 2021[242]). In chronic colitis, BBR alleviates inflammation by inhibiting M1 polarization via the AKT1/SOCS1/NF-κB pathway (Liu et al., 2018[113]; Luo et al., 2022[119]). Additionally, BBR competitively binds to the His224 site of trp53inp1 to inhibit macrophage pyroptosis, alleviating T-2 toxin-induced subacute liver injury (Xu et al., 2024[226]). BBR also reduces macrophage infiltration to ameliorate NASH (Wang et al., 2021[198]). In metabolic and neurological disorders, BBR improves obesity-related metabolic dysfunction by suppressing M1 polarization (Noh et al., 2022[136]) and promotes microglial M2 polarization through TYRO Protein Tyrosine Kinase Binding Protein (TYROBP) activation and SOCS1 upregulation, enhancing amyloid-beta peptide (Aβ) clearance and attenuating neuroinflammation and cognitive decline in Alzheimer's disease (AD) (Guo et al., 2021[48]; Yang et al., 2024[243]).

#### Regulation of Other Types of Immune Cells

Regulatory T cells (Treg cells) are a subset of T lymphocytes with immunosuppressive functions that prevent excessive immune responses and the development of autoimmune diseases. BBR exerts therapeutic effects by modulating the function of Treg cells. It enhances Treg cells activity, reduces inflammation, and improves intestinal injury and survival in septic mice. BBR also suppresses macrophage activation via Cytotoxic T-Lymphocyte-Associated Protein 4 (CTLA-4) mediated cell-cell contact by Treg cells, thereby reducing pro-inflammatory cytokine release and alleviating sepsis-induced liver injury (Qiu et al., 2022[148]). In NASH, BBR alleviates hepatic inflammation by increasing the Treg/T helper cell 17 (Th17) ratio and regulating the chemerin/Chemokine-Like Receptor 1 (CMKLR1) signaling pathway to reduce lipid accumulation (Lu et al., 2021[117]).

BBR also regulates other types of immune cells to exert therapeutic effects. In hepatocellular carcinoma (HCC), BBR decreases the proportion of effector CD8⁺ T lymphocytes expressing high levels of inhibitory receptors while increasing central memory CD8⁺ T cells with potential antitumor activity. BBR regulates antitumor immunity by modulating cytokine-mediated receptor-ligand interactions between immune cells (Hu et al., 2024[61]). In Helicobacter pylori-induced chronic gastritis, BBR also reduces the production of B cell-activating factors from DCs and CD4⁺ T cells, inhibits IL-17 production, and lowers the proportion of Th17 cells, exerting anti-inflammatory effects (Wu et al., 2018[214]). 

Additionally, BBR enhances the cytotoxicity of NK cells against HCC cells and reduces immune evasion by inhibiting IFN-γ-induced Programmed Death-Ligand 1 (PD-L1) expression, thereby exerting anti-HCC effects (Wang et al., 2022[185]).

### Maintenance of Cellular Homeostasis 

#### Regulation of Autophagy

**Figure 4:**
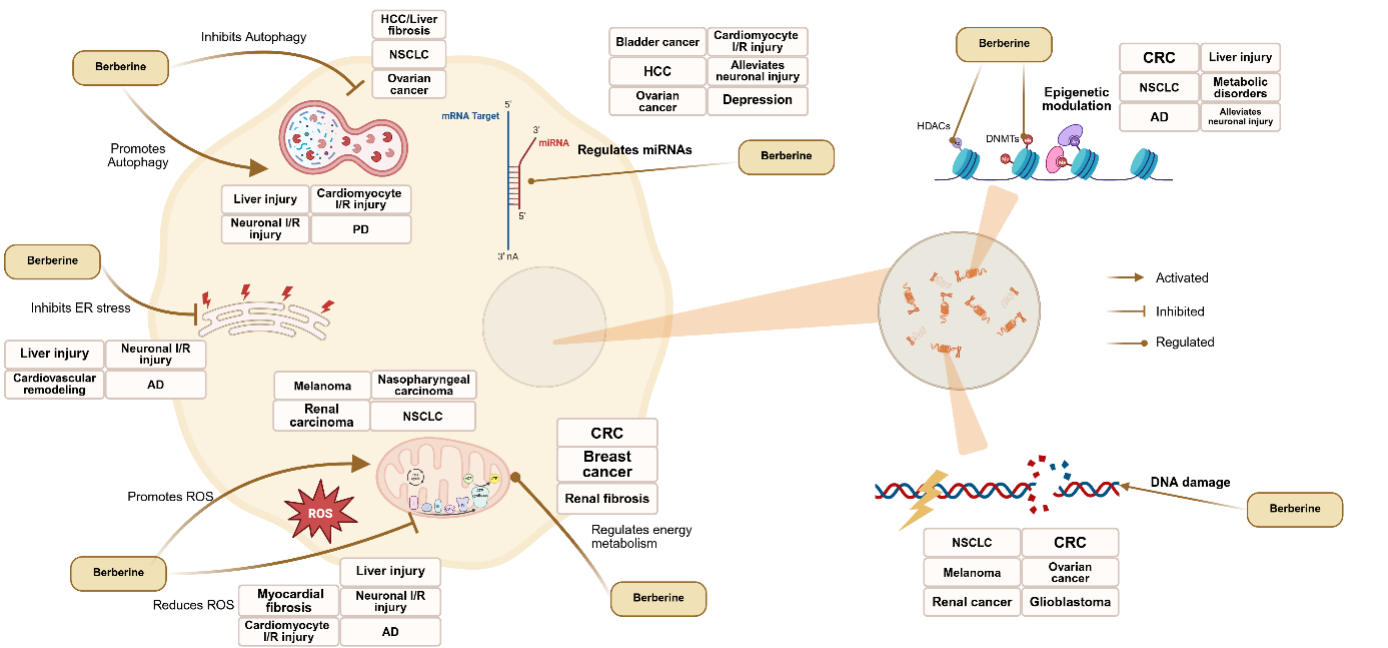
BBR influences the homeostasis and function of key effector cells across various diseases by regulating autophagy, oxidative stress, endoplasmic reticulum stress, and energy reprogramming, as well as by inducing DNA damage and modulating epigenetic mechanisms and non-coding RNAs. This figure was created by BioRender.com.

Autophagy, as a critical biological process for maintaining cellular homeostasis and adapting to stress, plays a key role in the development of various diseases. Increasing evidence indicates that BBR exerts therapeutic effects on multiple tumors and inflammation-related disorders by modulating autophagic activity and influencing cellular functions (Figure 4(Fig. 4)).

In gastrointestinal tumors, BBR suppresses mitochondrial complex I activity and induces mitophagy and ferroptosis via the parkin RBR E3 ubiquitin protein ligase (Parkin) / PTEN-induced putative kinase 1 (PINK1) pathway, significantly inhibiting tumor cell proliferation, invasion, and chemoresistance in vitro (Mori et al., 2023[130]). Conversely, in HCC, BBR achieves antitumor effects by inhibiting autophagy and inducing apoptosis and necrosis of cancer cells (Tai et al., 2020[173]). In ovarian cancer, BBR disrupts the interaction between LINC01123 and P65, reducing P65 protein stability and subsequently downregulating MAPK transcription, thereby modulating autophagy and inhibiting tumor cell proliferation and metastasis (Yan et al., 2024[232]). Moreover, combination therapy with BBR and erlotinib enhances antitumor efficacy by promoting autophagy-dependent EGFR degradation, thereby suppressing the proliferation of EGFR- tyrosine kinase inhibitor (TKI)-resistant non-small cell lung cancer (NSCLC) cells and reducing tumor growth in vivo (Chen et al., 2021[17]).

BBR activates the AMPK/mTOR pathway to promote autophagy, inhibits lysozyme secretion by Paneth cells, and attenuates colonic inflammation (Xu et al., 2022[225]). Similarly, in hyperglycemia-induced liver injury, BBR enhances autophagy via activation of the AMPK/mTOR pathway, alleviates lipid accumulation, suppresses inflammation and mitigates liver injury (Khater et al., 2023[78]). Meanwhile, BBR also induces hepatic stellate cells (HSCs) apoptosis by upregulating miR-30a-5p to inhibit autophagy-related 5 (ATG5) mediated autophagy, thereby reducing collagen deposition and inflammatory responses in mouse hepatic fibrosis models (Tan et al., 2023[174]). 

BBR alleviates hypoxia/reoxygenation (H/R)-induced excessive autophagy via the Rho family GTPase E (RhoE) /AMPK pathway, improving mitochondrial function and redox homeostasis in cardiomyocyte injury (Hu et al., 2024[59]). Additionally, BBR activates mitophagy through the PINK1/Parkin pathway to ameliorate myocardial damage in heart failure models (Abudureyimu et al., 2020[1]), and enhances Bcl-2 nineteen kilodalton interacting protein 3 (BNIP3) expression via HIF-1α-mediated transcriptional regulation, thereby protecting cardiomyocytes through mitophagy activation in myocardial ischemia (Zhu et al., 2020[271]). 

In cerebral ischemia model, BBR promotes autophagic flux by inducing transcription factor EB (TFEB) nuclear translocation for lysosome biogenesis and enhancing autophagosome-lysosome fusion via activation of the membrane fusion protein N-ethylmaleimide-sensitive factor (NSF), thus alleviating neuronal damage (Liu et al., 2024[114]; Zhuang et al., 2025[275]). In contrast, BBR also protects ischemia-reperfusion (I/R) induced neuronal injury by inhibiting autophagy through the mTOR pathway (Xie et al., 2020[219]). Additionally, in Parkinson's disease (PD) models, BBR enhances autophagy to suppress neuroinflammation, protect dopaminergic neurons, and improve behavioral impairments (Huang et al., 2021[66]).

These bidirectional regulatory effects of BBR on autophagy may depend on cell type, timing of intervention, and disease stage.

#### Effects on Oxidative Stress

Oxidative stress plays a key role in the pathogenesis of numerous diseases. BBR exerts bidirectional regulation of oxidative stress: it promotes reactive oxygen species (ROS) generation to enhance cytotoxicity against tumor cells, while concurrently reducing ROS accumulation to alleviate inflammation‐induced tissue damage (Figure 4(Fig. 4)).

In tumor models, BBR modulates the antioxidant response to promote ROS generation, thereby inhibiting melanoma cell proliferation and inducing apoptosis in vitro (Palma et al., 2022[139]). It also induces DNA damage in renal carcinoma cells via increased ROS accumulation, suppressing tumor progression and epithelial-mesenchymal transition (EMT) (Zhao et al., 2023[262]). 

BBR activates the AMPK and JNK signaling pathways and reduces ROS production by modulating the Nrf2-Kelch Like ECH Associated Protein 1 (Keap1)-antioxidant response element (ARE) antioxidant pathway and upregulating manganese superoxide dismutase (Mn-SOD). This suppresses oxidative stress-induced inflammation, inhibits p53-mediated hepatocyte apoptosis, protects liver cells, and mitigates liver injury (Han et al., 2019[51]; Gholampour et al., 2022[45]; Zhu et al., 2023[273]; Cheng et al., 2024[24]; Zhuang et al., 2024[274]). Additionally, during liver transplantation, the addition of BBR to the organ preservation solution has been shown to reduce I/R-induced lactate dehydrogenase (LDH) release, maintain the activity of mitochondrial complexes I and III in hepatocytes, decrease oxidative stress, and protect the donor liver (Martins et al., 2018[125]).

BBR attenuates oxidative damage and myocardial fibrosis by upregulating Nrf2 and activating the cytoprotective enzyme heme oxygenase-1 (HO-1) in doxorubicin (DOX)- and arsenic trioxide (ATO)-induced cardiotoxicity (Wang et al., 2023[196]; Hosseini et al., 2024[57]). BBR also induces the expression of miR-26b-5p, inhibits the activation of the MAPK signaling pathway, reduces reactive oxygen species (ROS) levels, thereby alleviating ventricular arrhythmias and infarct size caused by myocardial I/R injury, and improves cardiac function (Chen et al., 2021[11]; Jia et al., 2022[71]).

In the nervous system, BBR enhances antioxidant capacity, reduces oxidative stress and neuronal apoptosis, suppresses neuroinflammation, and thereby ameliorates cognitive impairment induced by HFD, AD, I/R injury, and hepatic encephalopathy (Hajipour et al., 2023[49]; Mehboodi et al., 2024[126]; Wu et al., 2024[210]). 

Moreover, BBR helps preserve blood-brain barrier integrity by reducing oxidative stress, decreases neuronal apoptosis in the hippocampal CA1 region, and mitigates I/R induced brain injury (Mehboodi et al., 2024[126]).

Ferroptosis is a critical consequence of oxidative stress, as excessive ROS production promotes lipid peroxidation, driving cells into ferroptosis. BBR modulates ferroptosis by affecting the balance between ROS generation and antioxidant defense, thereby influencing disease progression. In nasopharyngeal carcinoma, BBR induces ferroptosis by inhibiting the System Xc⁻/GSH/GPX4 axis, leading to increased ROS, lipid peroxidation, and Fe²⁺ accumulation (Wu et al., 2024[215]). In NSCLC, BBR synergizes with ferroptosis inducers to kill cancer cells via the p53-dependent solute carrier family 7 member 11 (SLC7A11)-GPX4 pathway, an effect absent in p53-mutant cells (Liao et al., 2024[105]). Additionally, BBR promotes ferritin degradation, resulting in Fe²⁺ overload and activation of ferroptosis, selectively eliminating activated HSCs and attenuating liver fibrosis (Yi et al., 2021[248]).

Conversely, BBR also exhibits cytoprotective effects in cardiovascular and neurodegenerative diseases by inhibiting ferroptosis. It activates the NRF2/SLC7A11/GPX4 pathway to suppress ferroptosis within atherosclerotic plaques, reducing lesion area and oxidative stress to protect vascular endothelial integrity (Wang et al., 2024[193]). In AD models, BBR upregulates GPX4 and SLC7A11 via NRF2, improves brain iron metabolism, inhibits neuronal ferroptosis, and ameliorates cognitive dysfunction (Li et al., 2023[100]).

#### Endoplasmic reticulum stress

Endoplasmic reticulum (ER) stress plays a critical role in maintaining cellular homeostasis, metabolic balance, and regulating inflammatory responses. Studies have shown that BBR alleviates protein misfolding and ER dysfunction by inhibiting ER stress and modulating the unfolded protein response (UPR), thereby exerting protective effects in inflammation-related diseases (Yarmohammadi et al., 2022[246]) (Figure 4(Fig. 4)).

BBR alleviates ER stress, suppresses inflammation, and improves liver injury induced by I/R, LPS, cholestasis, and hyperglycemia through downregulation of C/EBP homologous protein (CHOP) and activation of transcription factor 4 (TCF4) and X-box binding protein 1 (XBP1) (Zhang et al., 2017[258]; Wang et al., 2020[202]; Khater et al., 2023[78]; Wang et al., 2024[200]).

In myocardial infarction, BBR alleviates ER stress in cardiac tissue by upregulating Bcl-2/Bax expression and downregulating caspase-3, inhibiting cardiomyocyte apoptosis and improving cardiac remodeling (Liao et al., 2018[106]). It also suppresses the protein disulfide isomerase (PDI)-mediated ER stress pathway to reduce endothelial cell apoptosis and abnormal proliferation induced by mechanical stretch, thereby ameliorating hypertension-related vascular remodeling (Wang et al., 2020[186]).

Additionally, BBR inhibits canopy FGF signaling regulator 2 (CNPY2) and activates the mTOR pathway to attenuate ER stress and mitigate neuronal I/R injury (Xie et al., 2020[219]; Zhao et al., 2021[260]). In AD models, BBR reduces beta-site amyloid precursor protein cleaving enzyme 1 (BACE1) protein levels via the protein kinase RNA-like endoplasmic reticulum kinase (PERK)/eukaryotic translation initiation factor 2 alpha (eIF2α) axis, suppressing ER stress in neurons, decreasing Aβ-induced neuronal apoptosis, and improving cognitive function (Xuan et al., 2020[228]; Liang et al., 2021[104]).

#### Regulation of Cellular Energy Metabolism

BBR regulates metabolic reprogramming to inhibit the growth of various tumor cells. BBR inhibits mitochondrial complex I activity, which leads to the blockade of the electron transport chain, a reduction in ATP production, and suppression of energy metabolism in CRC cells, consequently limiting cell proliferation and promoting apoptosis (Wu et al., 2023[207]). In breast cancer, BBR effectively suppresses cell migration by inhibiting glycolysis, demonstrating notable anti-metastatic potential (Qian et al., 2021[143]; Du et al., 2022[32]). Moreover, BBR impairs the efflux function of ATP-binding cassette transporters and downregulates their expression, enhancing the sensitivity of breast cancer cells to DOX and reversing multidrug resistance (Qian et al., 2021[143]).

Furthermore, BBR restores mitochondrial energy homeostasis by activating PPARγ-coactivator-1α (PGC-1α), thereby improving oxidative phosphorylation in renal tubular cells, reducing the expression of mitochondrial fission protein dynamin-related protein 1 (Drp1), and enhancing mitochondrial biogenesis, which alleviates proteinuria and renal fibrosis in diabetic nephropathy (Qin et al., 2020[145]) (Figure 4(Fig. 4)).

### Regulation of DNA and Gene Expression by BBR

#### BBR-Induced DNA Damage

BBR exerts anti-tumor effects by inducing DNA damage or interfering with DNA repair mechanisms, leading to the suppression of tumor cell proliferation and the promotion of apoptosis. In NSCLC cells, BBR downregulates key proteins involved in DNA replication and repair, including ribonucleotide reductase regulatory subunit M1 (RRM1), RRM2, DNA ligase 1 (LIG1), and DNA polymerase epsilon subunit 2 (POLE2), thereby inhibiting cell proliferation (Li et al., 2018[95]; Ni et al., 2022[132]). Additionally, BBR increases ROS generation to induce DNA damage, reducing the viability of melanoma and renal cancer cells, resulting in triggering apoptosis (Palma et al., 2022[139]; Zhao et al., 2023[262]). BBR also enhances the efficacy of other therapies that induce DNA damage. It sensitizes ovarian cancer cells to radiotherapy by amplifying oxidative stress-induced DNA damage and apoptosis (Aleissa et al., 2023[2]). Co-administration with andrographolide inhibits DNA replication-related genes, suppresses proliferation, and induces cell cycle arrest in CRC cells (Zhao et al., 2022[263]). Similarly, combination therapy with solid lipid curcumin particles augments DNA damage and enhances glioblastoma cell death (Maiti et al., 2019[124]) (Figure 4(Fig. 4)).

#### Epigenetic Modulation by BBR

BBR exerts anti-tumor effects through epigenetic regulation, primarily by modulating DNA methyltransferases (DNMTs) and histone deacetylases (HDACs). BBR suppresses the proliferation of CRC cells by downregulating DNMT1 and DNMT3B, inhibiting the expression of the oncogene c-Myc (Wang et al., 2024[194]). In combination with evodiamine, BBR restores the expression of miR-152, miR-429, and miR-29a, affecting DNMTs activity and inhibiting the growth of CRC (Huang et al., 2017[62]). BBR acts similarly to a HDAC inhibitor, reactivating tumor suppressor genes such as p21 and p53 by increasing histone acetylation and downregulating oncogenes, ultimately inducing cell cycle arrest and apoptosis in NSCLC (Kalaiarasi et al., 2016[74]). Furthermore, combination BBR with metformin reduces the expression of specificity protein 1 (SP1) and 3-phosphoinositide-dependent protein kinase-1 (PDPK1), inhibits DNMT1 activity, and suppresses proliferation and migration of NSCLC (Zheng et al., 2018[265]).

In addition to its anti-tumor activity, BBR exerts systemic therapeutic benefits through epigenetic regulation. It alleviates Staphylococcus aureus enterotoxin B (SEB)-induced acute liver injury and inflammation by suppressing the expression of HDAC1 (Du et al., 2018[32]). BBR also promotes glucagon-like peptide-1 (GLP-1) secretion by intestinal L cells via activation of β-catenin/TCF4 signaling, which is mediated by increased methylation and downregulation of miR-106b, ultimately contributing to the improvement of metabolic disorders in HFD-fed mice (Wang et al., 2021[184]). In AD models, BBR restores mitophagy and alleviates D-ribose-induced cognitive deficits by inhibiting promoter methylation of PINK1 (Wang et al., 2023[181]). Furthermore, in stroke models, BBR enhances nuclear-enriched abundant transcript 1 (NEAT1) stability via methyltransferase-like 3 (METTL3)-mediated m6A modification, modulating the NEAT1/miR-377-3p/nicotinamide phosphoribosyl transferase (NAMPT) axis to exert neuroprotective effects (Hu et al., 2024[60]) (Figure 4(Fig. 4)). 

#### Regulation of non-coding RNAs by BBR

BBR regulates non-coding RNAs (ncRNAs) to interfere with the expression of key proteins and influence cellular functions, exerting therapeutic effects in various tumors as well as cardiovascular and neurological diseases (Figure 4(Fig. 4)). 

BBR inhibits the proliferation, migration, and invasion of bladder cancer cells and induces apoptosis by upregulating miR-17-5p and suppressing the JAK1-STAT3 signaling pathway (Xia et al., 2021[218]). It also inhibits the proliferation, migration, and invasion of ovarian cancer cells by upregulating miR-145 and downregulating MMP16 expression (Li et al., 2021[96]). In combination with curcumin, BBR regulates the miR-221/ SRY-box transcription factor 11 (SOX11) axis to activate pro-apoptotic proteins caspase-3/9, promoting apoptosis in HCC cells and exerting anti-hepatocarcinoma effects (Li et al., 2023[98]).

In myocardial I/R injury, BBR alleviates myocardial cell apoptosis and inflammation by downregulating miR-184 and inhibiting the NOTCH1 signaling pathway, as well as upregulating miR-340-5p to inhibit high mobility group box 1 (HMGB1)-mediated TLR4/NF-κB activation, which contributes to the improvement of cardiac function (Long et al., 2022[115]; Yang et al., 2025[234]). In the nervous system, BBR protects neurons in AD by mitigating Aβ-induced neuronal apoptosis via the miR-188/ nitric oxide synthase 1 (NOS1) axis (Chen et al., 2020[16]). It also alleviates neuronal injury in PD by inhibiting the LINC00943/miR-142-5p/karyopherin subunit alpha 4 (KPNA4) axis and suppressing the NF-κB pathway (Li et al., 2021[101]). Additionally, BBR downregulates miR-34a, miR-34b-5p, and miR-470-5p in the mouse hippocampus, while significantly upregulating brain-derived neurotrophic factor (BDNF), synaptotagmin-1, and Bcl-2, improving dendritic spine morphology and promoting hippocampal neuron growth to relieve depressive symptoms (Yi et al., 2020[249]; Zhan et al., 2021[255]).

## Modulation of Cellular Functions by BBR

**Figure 5:**
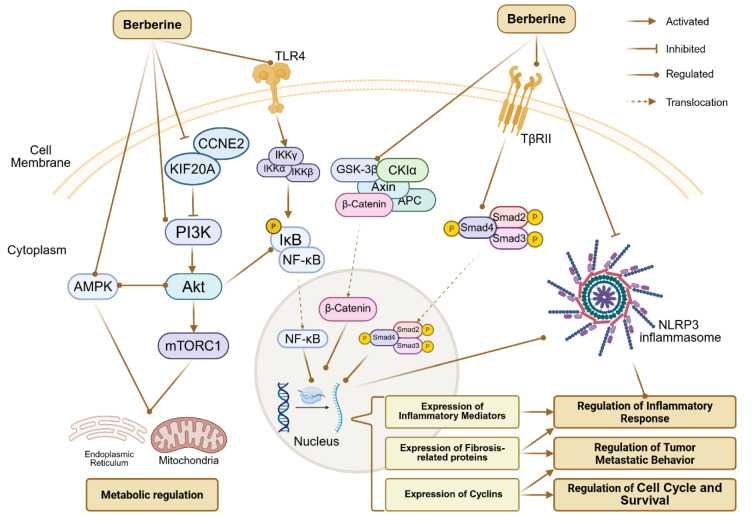
Key signaling pathways affected by BBR and their downstream effects. This figure was created by BioRender.com.

The systemic regulatory effects of BBR lead to functional changes in key effector cells across various diseases. In oncological conditions, BBR exerts anti-tumor effects by inhibiting tumor cell proliferation, migration, and invasion, while promoting apoptosis (Sun et al., 2022[168], 2023[167]). In inflammation-related disorders, BBR mitigates tissue damage and reduces apoptosis in effector cells such as cardiomyocytes and neurons by attenuating inflammatory responses (Huang et al., 2021[66]; Tang et al., 2024[176]). Through modulation of key signaling pathways such as PI3K/AKT, NF-κB, and Wnt/β-catenin, as well as regulation of the NOD-like receptor family pyrin domain containing 3 (NLRP3) inflammasome, BBR contributes to functional remodeling of effector cells and underlies its therapeutic potential in cancer, digestive, metabolic, neurological, and cardiovascular diseases (Figure 5(Fig. 5)).

### PI3K/Akt/mTOR Signaling Pathway

BBR exerts anti-proliferative and pro-apoptotic effects on tumor cells in various cancers by modulating the PI3K/AKT/mTOR signaling pathway. In NSCLC, BBR targets kinesin family member 20A (KIF20A) and cyclin e2 to inhibit the PI3K/AKT pathway, thereby suppressing cell proliferation, invasion, and glycolysis, while promoting apoptosis (Wang et al., 2023[191]). Similarly, BBR, in combination with low-temperature plasma (LTP), synergistically inhibits PI3K/AKT signaling and enhances apoptosis in NSCLC cells (Lu et al., 2023[116]).

In gastrointestinal malignancies, BBR enhances the sensitivity of CRC cells to heat shock protein 90 (HSP90) and HDAC inhibitors via modulation of the PI3K/AKT pathway (Li et al., 2021[89]). Moreover, co-administration of BBR and oligomeric proanthocyanidins (OPC) suppresses the MYB/PI3K-AKT pathway and upregulates apoptosis-related genes (Okuno et al., 2022[137]).

In other cancers, BBR downregulates RAD51 recombinase via the PI3K/AKT pathway, enhancing gemcitabine cytotoxicity and improving its therapeutic efficacy against bladder cancer (Gao et al., 2021[40]). BBR combined with solid lipid curcumin particles (SLCP) is more effective than monotherapy in reducing PI3K/AKT/mTOR pathway activity and inducing apoptosis in glioma cells (Maiti et al., 2019[124]).

Beyond oncology, BBR also regulates cellular function and inflammatory response through the PI3K/AKT/mTOR pathway. BBR, in combination with curcumin (CUR), alleviates acetaminophen (APAP)-induced hepatic inflammation in mice by inhibiting the PI3K/AKT signaling pathway (Zhai et al., 2024[254]). BBR also suppresses the PI3K/AKT pathway, downregulates proinflammatory cytokines (such as IL-8 and TNF-α), and activates autophagy to ameliorate gastric mucosal lesions in chronic atrophic gastritis (CAG) (Tong et al., 2021[177]). Additionally, in myocardial I/R injury, BBR pretreatment inhibits the PI3K/AKT pathway and its downstream inflammatory mediators (IL-6, IL-1β), thereby reducing the incidence of arrhythmia and protecting cardiomyocytes (Qin-Wei and Yong-Guang 2016[147]).

In cerebral I/R injury, BBR reduces neuronal apoptosis and attenuates brain injury via the BDNF, tropomyosin receptor kinase B (TrkB) and PI3K/AKT pathway (Yang et al., 2018[235]). It also mitigates hippocampal neuronal apoptosis and damage through PI3K/AKT signaling, alleviating depressive symptoms and improving cognitive function in mice (Wei et al., 2023[205]; Tang et al., 2024[176]). Furthermore, BBR protects dopaminergic neurons through anti-apoptotic, anti-inflammatory, and antioxidant mechanisms via the PI3K/AKT pathway, thereby ameliorating behavioral deficits in a PD mouse model (Wen et al., 2022[206]). 

### NF-κB Signaling Pathway

BBR suppresses NSCLC cell proliferation and promotes apoptosis by inhibiting the NF-κB pathway and downregulating activator protein-1 (AP-1) expression (Li et al., 2018[95]). BBR also alleviates ulcerative colitis (UC) by targeting the TLR4/NF-κB pathway to inhibit IL-6, AP-1, and HIF-1α, while upregulating anti-inflammatory cytokines IL-4 and IL-10 to suppress the inflammatory response (Zhu et al., 2019[270]; Li et al., 2020[85]; Xu et al., 2020[227]; Li et al., 2024[93]). It also relieves hippocampal neuronal pyroptosis in rats with cerebral I/R injury via upregulation of PPAR-γ and inhibition of NF-κB signaling (Zhao et al., 2021[261]). In an in vitro AD model induced by Aβ in HT22 cells, BBR reduces oxidative stress and apoptosis through NF-κB inhibition (Zhang et al., 2023[259]). Similarly, in a methamphetamine (METH) withdrawal model, BBR alleviates anxiety-like behaviors and relapse while protecting hippocampal neurons by modulating NF-κB signaling (Rezaeian et al., 2020[152]). 

### Wnt/β-Catenin Signaling Pathway

BBR regulates the proliferation, apoptosis, and biological behavior of various tumor cells through modulation of the Wnt/β-catenin signaling pathway. In HCC, BBR promotes the binding of Eukaryotic translation initiation factor 4E-binding protein 1 (4E-BP) to the translation initiation complex, thereby inhibiting cap-dependent translation of β-catenin and reducing its protein levels, ultimately inducing apoptosis and suppressing the survival of HCC cells (Vishnoi et al., 2021[180]). In CRC, bioinformatics and experimental validation have shown that BBR induces the translocation of β-catenin from the nucleus to the cytoplasm, thereby inhibiting CRC cell proliferation-findings consistent with the elevated expression of catenin beta 1 mRNA in CRC patients and its association with advanced clinical stages (Nie et al., 2022[135]). Further studies revealed that BBR suppresses the epigenetic regulation of the Wnt/β-catenin pathway by downregulating the long intergenic ncRNA regulator of reprogramming (lincROR), leading to inhibited CRC growth both in vitro and in vivo (Li et al., 2023[99]). Additionally, BBR inhibits breast cancer cell migration and invasion by suppressing Wnt/β-catenin signaling and reversing EMT, while inducing cell cycle arrest at the G0/G1 or G2/M phase (Dian et al., 2022[29]).

### TGF-β/Smad Signaling Pathway

BBR inhibits tumor cell metastasis and EMT by modulating the transforming growth factor beta (TGF-β) signaling pathway. In a cecal implantation mouse model, BBR significantly reduced the number of hepatic metastatic lesions from CRC by upregulating E-cadherin, downregulating vimentin and Snail, and decreasing serum inflammatory cytokine levels-an effect comparable to that of TGF-β inhibitors (Kang et al., 2024[75]). In the tumor microenvironment of CRC, BBR also reverses tumor microenvironment-induced EMT-like phenotypic changes by regulating the expression of TβRII, Smad2, and phosphorylated Smad3 (p-Smad3), with a mechanism similar to that of TGF-β receptor antagonists (Huang et al., 2019[64]). In glioma, BBR suppresses cell migration and invasion by downregulating TGF-β1-mediated expression of collagen type XI alpha 1 (COL11A1) and matrix metalloproteinases (MMPs) (Sun et al., 2022[171]).

### AMPK Signaling Pathway

BBR plays a pivotal role in both oncological therapy and intestinal mucosal protection via modulation of the AMPK pathway. Multi-omics analyses and xenograft studies demonstrated that BBR downregulates RRM2 expression by inhibiting HIF-1α and AMPK signaling, thereby blocking EMT and suppressing breast cancer cell migration and invasion (He et al., 2022[54]). In a colitis-induced mucosal barrier injury model, BBR mitigates tight junction disruption by an AMPK-dependent downregulation of swiprosin-1, which in turn inhibits myosin light chain kinase (MLCK) -mediated cytoskeletal contraction in colonic epithelial cells (Wang et al., 2024[204]).

BBR can also suppress inflammatory responses by inhibiting the AMPK signaling pathway, downregulating inflammatory cytokines such as IL-6, IL-8, IFN-γ and TNF-α and upregulating IL-10, thereby suppressing inflammatory responses and alleviating liver injury (Wang et al., 2017[201]; Luo et al., 2019[118]), and myocardial I/R injury (Chang et al., 2012[10], 2016[9]).

### NLRP3 inflammasome

The NLRP3 inflammasome is a multiprotein complex that plays a pivotal role in regulating inflammatory signaling. In various inflammatory diseases, aberrant activation and oligomerization of NLRP3 lead to the activation of caspase-1 and the release of multiple cytokines (Blevins et al., 2022[7]). BBR exhibits significant anti-inflammatory effects in hepatic and neurological disease models by inhibiting NLRP3 activation. In liver injury models, BBR targets NLRP3 to suppress caspase-1 activation, reduce ROS production and ECM accumulation, thereby alleviating drug-induced and acute liver injury (Ali and Datusalia 2024[4]; Zhuang et al., 2024[274]). In the nervous system, BBR enhances autophagic activity to inhibit 1-methyl-4-phenyl-1,2,3,6-tetrahydropyridine (MPTP) -induced NLRP3 inflammasome activation, protect dopaminergic neurons, mitigate neuroinflammation in PD models, and improve motor dysfunction (Huang et al., 2021[66]). BBR also promotes Trim65-mediated ubiquitination and degradation of NLRP3, alleviates hippocampal neuronal dysfunction, and restores synaptic plasticity and neurogenesis (Qin et al., 2023[146]; Yang et al., 2023[237]). Moreover, in cerebral ischemia models, BBR suppresses NLRP3 to reduce middle cerebral artery occlusion (MCAO) -induced neurological deficits, neuroinflammation, and blood-brain barrier damage, exhibiting marked neuroprotective effects (Rahman et al., 2024[149]).

### Other Signaling Pathway

BBR exerts anti-CRC effects by modulating the Hedgehog signaling pathway. Specifically, BBR suppresses malignant phenotypes and induces apoptosis in CRC cells through inhibition of Hedgehog signaling, leading to cell cycle arrest and impaired tumor cell proliferation. Notably, these effects are selective to cancer cells, as BBR demonstrates no cytotoxicity toward normal colonic epithelial cells (Sun et al., 2022[168], 2023[167]).

In other organs, BBR suppresses the proliferation and metastasis of gastric cancer cells by inhibiting the IL-6/JAK2/STAT3 pathway (Xu et al., 2022[224]). Moreover, co-administration of BBR with erlotinib synergistically inhibits EGFR /AKT signaling, significantly enhancing cytotoxicity against EGFR-positive human epidermoid carcinoma cells in vitro, thereby offering a potential strategy to overcome EGFR-TKI resistance (Cuan et al., 2023[26]). Additionally, BBR promotes intestinal epithelial regeneration in radiation enteritis by enhancing intestinal stem cell (ISC) function via activation of the STAT3/ERK1/2 signaling axis (Tu et al., 2024[178]).

Separately, Jiao-tai-wan, with BBR as its principal active component, protects neuronal cells by activating the cyclic adenosine monophosphate (cAMP) / protein kinase A (PKA) / cAMP response element-binding protein (CREB) signaling pathway, alleviating glucose and lipid metabolic disturbances as well as depression-like behaviors in diabetic mice with comorbid depression (Tang et al., 2024[175]).

## Regulation of Gut Microbiota by BBR

The gut microbiota serves as a key interface between the host and the environment. Beyond the direct effects on the host, BBR modulates the gut microbiota and its metabolites to exert systemic regulatory actions indirectly. 

Community analysis based on 16S rRNA gene sequencing revealed that BBR increased the abundance of Firmicutes, while decreasing the numbers of Bifidobacteria, Streptococci, and Enterococci. BBR also affects the metabolism of short-chain fatty acids (SCFA) such as acetate, propionate, and butyrate. Metabolomic analysis further indicated that BBR treatment regulated multiple amino acid metabolic pathways in the gut microbiota, particularly those involving tyrosine, serine, and L-glutamate (Fu et al., 2022[39]).

### Modulating gut microbiota structure

BBR delays the progression of CRC by modulating gut microbiota composition. Deng (Deng et al., 2022[28]) and Chen (Chen et al., 2020[14]), using Azoxymethane (AOM)/DSS-induced mouse models, found that berberine increased the Firmicutes-to-Bacteroidetes (F:B) ratio, promoted the growth of SCFA-producing bacteria such as Alloprevotella, and reduced pro-inflammatory bacteria such as Alistipes, thereby significantly reducing colonic tumor numbers and improving intestinal inflammation. Berberine also decreased the abundance of the pro-carcinogenic bacterium Veillonella parvula, thereby weakening B cell immunoregulatory functions such as the BLyS signaling pathway and delaying CRC development (Qian et al., 2023[144]). 

BBR exerts protective effects against colonic injury and colitis primarily through modulation of the gut microbiota. It increases the abundance of beneficial bacteria such as lactic acid bacteria, carbohydrate-hydrolyzing bacteria,* Akkermansia*, *Lactobacillus spp.*, and *Parabacteroides*, while reducing opportunistic pathogens including *Bacteroides acidifaciens* and *Bacteroides fragilis*, which enhances the expression of epithelial tight junction proteins such as zonula occludens-1 (ZO-1) and occluding, reduces the Th17/Treg ratio, maintains the structural and functional integrity of the intestinal barrier, and regulates mucosal immune homeostasis, thereby alleviating colonic inflammation and mucosal injury (Liao et al., 2020[107]; Zheng et al., 2021[264]; Dong et al., 2022[31]; Li et al., 2022[102]; Yu et al., 2024[250]; Du et al., 2025[33]). Compound preparations containing berberine hydrochloride also ameliorate colitis by modulating the colonic microbiota, particularly pathways related to bacterial DNA synthesis, replication, and repair (Yan et al., 2022[233]; Xu et al., 2023[221]; Zhou et al., 2023[268]). 

In metabolic diseases, BBR reshapes the gut microbiota structure, restores microbial diversity, and upregulates intestinal GLP-2 secretion to slow the progression of diabetes (Wang et al., 2021[197]). The combination of BBR and metformin enhances insulin sensitivity by altering microbiota composition, such as enriching the phylum *Verrucomicrobia *(Lyu et al., 2022[121]). BBR also improves uric acid metabolism by increasing the abundance of *Lactobacillus* and reducing *Bacteroidetes *(Chen et al., 2023[19]). Additionally, a randomized controlled trial showed that 12-week BBR treatment significantly improved IR and dyslipidemia in patients treated with olanzapine, and reduced the F:B ratio (Pu et al., 2021[141]).

BBR improves the progression of cardiovascular diseases by modulating the gut microbiota. BBR increases the *Firmicutes / Verrucomicrobia* ratio, reduces hepatic flavin-containing monooxygenase 3 (FMO3) expression and serum trimethylamine N-oxide (TMAO) levels, thereby alleviating atherosclerotic plaque formation in HFD-mice (Shi et al., 2018[158]). Additionally, BBR inhibits CutC/D enzyme activity in gut microbiota, reducing TMAO production and subsequently mitigating endoplasmic reticulum stress-mediated endothelial cell apoptosis, improving vascular dysfunction, and alleviating hypertension in mice (Wang et al., 2024[203]).

In neurological diseases, BBR improves gut microenvironment by increasing beneficial bacteria such as *Akkermansia*, enhancing intestinal barrier repair and suppressing activation of the gut-brain axis inflammatory pathway, reducing Aβ deposition and inhibiting BACE1 expression, which significantly improves cognitive function in AD mouse models (Sun et al., 2024[165]). BBR also enriches enterolignan-producing bacteria such as *Bacteroides* and *Bifidobacterium*, elevates serum enterolignan levels, and markedly alleviates anxiety-like behaviors (Fang et al., 2021[38]). Furthermore, BBR alters gut microbiota composition, reducing pro-inflammatory bacteria such as *Muribaculaceae* and increasing *Bacteroidaceae* to inhibit neuronal ferroptosis in mice with I/R injury and mitigate cerebral ischemic damage (Wang et al., 2023[195]).

### Regulation of metabolic products

SCFA are the primary end-products of non-digestible carbohydrate (NDC) fermentation and serve as key metabolites utilized by the gut microbiota. They represent a major flow of carbon from diet to microbiota and then to the host, with major products including formate, acetate, propionate, and butyrate (Morrison and Preston 2016[131]). BBR promotes the production of SCFAs such as butyrate and acetate by enhancing the growth of SCFA-producing bacteria like *Alloprevotella *(Chen et al., 2020[14])*, Blautia producta *(Yang et al., 2022[244]) and* Roseburia *(Wu et al., 2020[211]), facilitating mucosal barrier repair and suppressing inflammation (Li et al., 2022[94]; Yan et al., 2022[230]). Specifically, butyrate inhibits CRC growth by suppressing HDAC1 expression (Huang et al., 2022[63]), attenuates HCC growth by reducing PPARδ degradation (Shou and Shaw 2023[159]), and alleviates post-stroke neurological dysfunction by elevating serum butyrate levels, thereby inhibiting microglial and astrocyte activation and reducing the release of pro-inflammatory cytokines (Duan et al., 2023[35]). Additionally, BBR-induced increase in SCFA production can improve hyperlipidemia in HFD-induced mice (Yang et al., 2022[244]), and upregulate hippocampal neurotransmitters and BDNF expression, thereby alleviating depression-like behaviors in stress-exposed rats (Huang et al., 2023[65]). 

BBR influences the progression of various diseases by modulating specific amino acid metabolites derived from the gut microbiota. It activates the aryl hydrocarbon receptor (AhR) via microbial tryptophan metabolites, thereby restoring intestinal barrier integrity, reducing the incidence of colorectal tumors (Wang et al., 2024[188]) and alleviating colitis (Jing et al., 2021[72]; Chen et al., 2023[21]). Additionally, BBR enhances the expression of tryptophan hydroxylase 1 (TPH1) and indoleamine 2,3-dioxygenase 1 (IDO1) through microbiota-mediated metabolic regulation, promoting the conversion of tryptophan to serotonin while inhibiting the kynurenine pathway, thus improving depression-like behavior in chronically stressed mice (Ge et al., 2023[43]). The improvement of glucose and lipid metabolic disorders in mice by BBR is also associated with reduced levels of isoleucine and phenylalanine derived from gut microbial metabolism (Fang et al., 2022[37]).

BBR alleviates the progression of UC, NASH, and hyperlipidemia by modulating bile acid metabolism mediated by the gut microbiota. BBR restores microbial balance, increases gastrointestinal unconjugated and secondary bile acid levels, activates the FXR and G protein coupled bile acid membrane receptor 5 (TGR5) signaling pathways to improve colonic inflammation (Sun et al., 2023[170]), and promotes intestinal mucosal barrier repair by inhibiting the S1PR2/RhoA/ROCK signaling pathway (Yu et al., 2024[251]). BBR also increases the abundance of *Clostridiales* and *Lactobacillaceae*, suppresses *Clostridium* species, promotes bile acid deconjugation, reduces the hydrophobicity of secondary bile acids, activates the intestinal FXR/FGF15 axis, and thereby decreases cholesterol absorption and hepatic lipid accumulation, ultimately improving NASH and hyperlipidemia (Shu et al., 2021[160]; Wang et al., 2024[190]).

BBR also regulates the progression of various diseases by modulating other microbiota-derived metabolites. BBR directly targets the FtfL enzyme of the pathogenic bacterium *Peptostreptococcus anaerobius*, disrupting its tetrameric conformation and ATP binding, thereby inhibiting its pro-carcinogenic activity (Yan et al., 2023[229]). Additionally, BBR acts as a non-competitive inhibitor of bacterial β-glucuronidase (GUS), significantly reducing the intestinal accumulation of SN38, a toxic metabolite of irinotecan (CPT11), thus mitigating chemotherapy-induced mucositis without compromising the anticancer efficacy of CPT11 (Yue et al., 2021[253]). BBR also upregulates specific microbial taxa and promotes the production of phenolic lipids, thereby enhancing the intestinal barrier and alleviating colitis (Wu et al., 2025[212]). In metabolic diseases, BBR improves hepatic lipid accumulation and fibrosis in NAFLD mice by inhibiting fatty acid synthase via modulation of *Bacteroidaceae *(Li et al., 2022[91]). In the nervous system, BBR enhances dopamine levels in the brain and improves neurological function by promoting L-DOPA synthesis through *Enterococcus* species (Wang et al., 2021[199]). It also promotes microbial hydrogen sulfide (H₂S) production, activating the vagal transient receptor potential cation channel subfamily V member 1 (TRPV1) receptor and modulating microglial polarization, thereby alleviating neuroinflammation following cerebral ischemia (Ni et al., 2022[133]).

## Potential Toxicity and Clinical Safety of Berberine

BBR exerts anti-tumor effects by inhibiting cell proliferation and inducing apoptosis (Singh and Sharma 2018[162]), and it also improves metabolic disorders such as diabetes by suppressing gluconeogenesis (Moreira et al., 2022[129]). However, these therapeutic effects have raised concerns regarding the potential toxicity of BBR. Some studies have reported that high doses or prolonged exposure to BBR may induce DNA damage, mitochondrial dysfunction, and ion channel abnormalities (Singh and Sharma 2018[162]; Moreira et al., 2022[129]; Liu et al., 2024[111]). Importantly, these toxic effects are typically observed at doses far exceeding the clinically recommended range (0.5-1.5 g/day) (Ju et al., 2018[73]; Nie et al., 2024[134]). 

As a traditional Chinese medicine widely used for treating intestinal infections, BBR has been administered clinically for decades in China, and its safety profile has been extensively validated through long-term use (Gao et al., 2020[42]). Current clinical studies suggest that BBR is generally well tolerated, with adverse effects mainly limited to mild gastrointestinal discomfort (e.g., diarrhea, bloating), occurring in approximately 5%-10% of patients. No serious adverse events have been reported (Ye et al., 2021[247]; Nie et al., 2024[134]). Nevertheless, BBR may inhibit cytochrome P450 enzymes and thus affect the metabolism of other drugs. Therefore, caution and close monitoring are advised in patients with hepatic or renal impairment or those on multiple medications (Ali et al., 2021[3]; Shi et al., 2025[157]).

## Potential Targets of BBR

**Figure 6:**
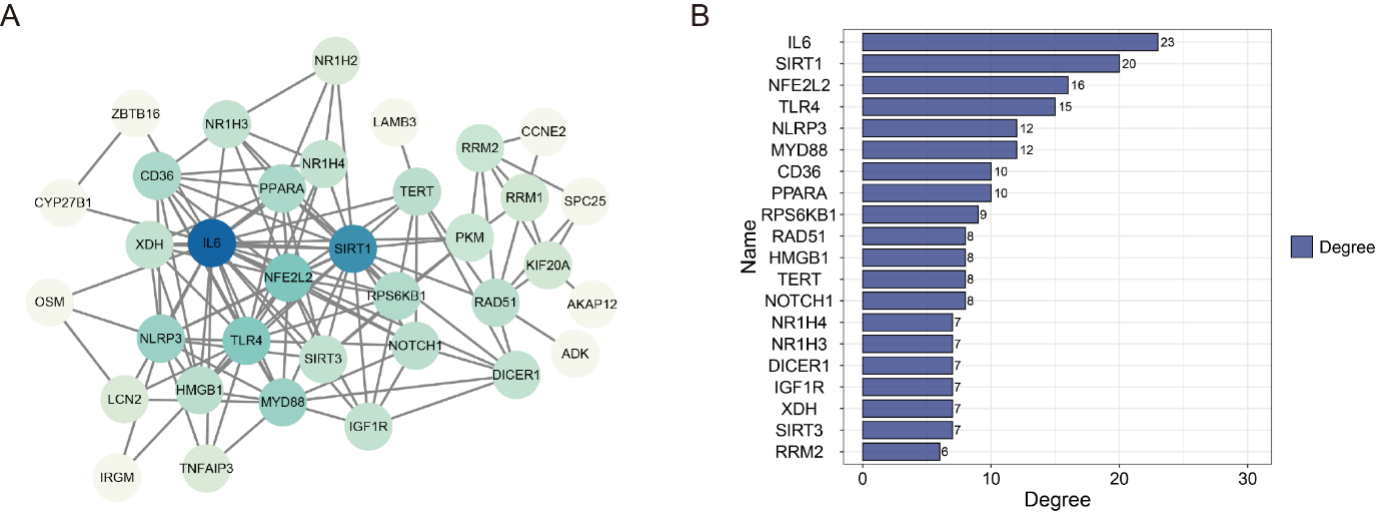
Potential Targets of BBR. (A) PPI network analysis on BBR protein targets. (B) The top 20 hub proteins in PPI network analyzed by Cytoscape.

To further elucidate the potential targets of BBR, we summarized key molecules identified in the literature that interact with BBR, including ncRNAs, enzymes, cytokines, receptors, and other types of proteins (see Table 1(Tab. 1) for details; References in Table 1: Ali and Datusalia 2024[4]; Cao et al., 2020[8]; Chen et al., 2023[19]; Cheng et al., 2024[24]; Clark et al., 2024[25]; Ding et al., 2023[30]; Gao et al., 2021[40]; Guan et al., 2020[47]; Hameed et al., 2024[50]; He et al., 2022[54]; He et al., 2023[53]; He et al., 2024[56]; Hou et al., 2019[58]; Huang et al., 2021[66]; Huang et al., 2024[67]; Jia et al., 2022[71]; Ke et al., 2022[76]; Li et al., 2018[88]; Li et al., 2020[85]; Li et al., 2020[90]; Li et al., 2021[89]; Li et al., 2022[86]; Li et al., 2023[99]; Liu et al., 2020[110]; Liu et al., 2022 [112]; Long et al., 2022[115]; Lv et al., 2025[120]; Meng et al., 2024[127]; Qin et al., 2023[146]; Samad et al., 2021[154]; Shaker et al., 2021[156]; Sun et al., 2024[166]; Wang et al., 2020[186]; Wang et al., 2023[191]; Wang et al., 2023[196]; Wu et al., 2018[216]; Wu et al., 2020[213]; Wu et al., 2024[209]; Xu et al., 2022[224]; Xu et al., 2024[220]; Yan et al., 2022[231]; Yang et al., 2021[236]; Yang et al., 2023[237]; Yao et al., 2023[245]; Zhang et al., 2023[259]; Zhong et al., 2023[266]; Zhu et al., 2018[269]). Subsequently, we conducted a protein-protein interaction (PPI) network analysis of the protein targets using the STRING database and found that most of them are functionally interconnected (Figure 6A(Fig. 6)). Degree ranking performed via Cytoscape revealed IL-6, SIRT1, and nuclear factor erythroid 2-related factor 2 (NFE2L2) as the top three hub proteins, suggesting they may serve as core mediators of BBR's pharmacological effects (Figure 6B(Fig. 6)).

## Clinical Applications of BBR

Clinical studies have confirmed the therapeutic efficacy of BBR in NAFLD, diabetes, and hyperlipidemia. 

A meta-analysis (including 10 RCTs with 811 patients) demonstrated that BBR significantly reduces liver enzymes (ALT, AST, GGT), blood lipids (TG, TC, LDL-C), and IR index, with only mild gastrointestinal adverse effects (Nie et al., 2024[134]). A double-blind RCT (n = 70) further showed that 12-week treatment with BBR (1500 mg/day) significantly reduced ALT, AST, and the ALT/AST ratio (p < 0.01), although improvements in other metabolic parameters were limited (Koperska et al., 2024[81]). For prediabetic patients, BBR (500 mg TID) significantly decreased fasting plasma glucose (FPG), hemoglobin A1c (HbA1c), and IR index to normal ranges (Panigrahi and Mohanty 2023[140]). Interestingly, BBR's lipid-regulating effects exhibited sex differences: it increased HDL-C in females but showed no significant effect in males (Blais et al., 2023[6]).

Moreover, a phase II trial of HTD1801 (a berberine-ursodeoxycholic acid compound) demonstrated significant reductions in liver fat content and improvements in glycemic control in patients with NASH and diabetes (Harrison et al., 2021[52]), and it also significantly decreased ALP levels in patients with primary sclerosing cholangitis (PSC) (Kowdley et al., 2022[82]). In H. pylori eradication therapy, an open-label RCT (n = 612) revealed that a BBR-containing quadruple regimen achieved a non-inferior eradication rate (90.1%) compared to a bismuth-based regimen (86.4%), with similar incidence of adverse events (Zhang et al., 2017[256]). However, a meta-analysis on hypertension indicated that evidence for BBR's blood pressure-lowering effect is limited and of low quality, and its combination with antihypertensive drugs (e.g., amlodipine) did not show additional benefit (Suadoni and Atherton 2021[164]).

## Strategies to Improve Bioavailability

Due to its low solubility, limited intestinal absorption, and first-pass metabolism, BBR exhibits poor oral bioavailability. To overcome these limitations, various strategies have been developed, primarily including nanotechnology-based delivery systems and structural modifications.

### Nanotechnology-based delivery systems

Organic nanocarriers are commonly used in BBR delivery systems due to their good biocompatibility and biodegradability. Polymeric nanoparticles encapsulate BBR within polymer matrices, protecting it from degradation in the gastrointestinal environment and enabling sustained release to enhance absorption (Cui et al., 2018[27]; Ghobadi-Oghaz et al., 2022[44]; Gao et al., 2023[41]). In models of breast cancer (Ghobadi-Oghaz et al., 2022[44]; Wu et al., 2024[208] ), colitis (Yang et al., 2022[239]; Li et al., 2024[92]), diabetes (Cui et al., 2018[27]), and AD (Saleh et al., 2024[153]), polymer-encapsulated BBR microspheres have demonstrated superior bioavailability and targeting capability compared to free BBR, resulting in improved therapeutic efficacy.

Liposomes are also a common type of nanocarrier that can enhance the solubility of poorly soluble herbal compounds and improve therapeutic efficacy in vivo (Jia et al., 2019[70]). Liposome-based BBR delivery systems (LipoNio.BBR) have demonstrated significantly improved organ-targeting ability and therapeutic effects compared to free BBR in diseases such as CRC (Ibrahim et al., 2023[68]; Mianowska et al., 2023[128]), liver cancer (Qi and Liu 2021[142]), lung cancer (Alnuqaydan et al., 2022[5]; Uma Maheswari et al., 2023[179]), colitis (Sun et al., 2023[170]), NAFLD (Chen et al., 2021[22]), and AD (Raju et al., 2021[150]; Wang et al., 2022[187]).

Inorganic nanocarriers, composed of inorganic materials, offer excellent stability, biocompatibility, and low cytotoxicity (Javed Iqbal et al., 2021[69]). Janus gold mesoporous silica nanocarriers, silver nanoparticles, and selenium nanoparticles have been shown to induce apoptosis in HCC more effectively than free BBR (Li et al., 2019[103]; Khaled et al., 2024[77]). Moreover, in a mouse model of Ehrlich solid tumors, selenium nanoparticles significantly reduced tumor volume and improved survival rates (Othman et al., 2022[138]).

### Structural modifications

As an organic heterocyclic compound, BBR's pharmacokinetic properties and pharmacological activity can be improved through chemical modifications at specific carbon sites, with the C-9 and C-13 positions being the most frequently targeted. For instance, 9/13-O-dodecyl BBR improves the photocytotoxicity of BBR against HCC (Lin et al., 2020[108]); 13-butoxyberberine enhances its anti-migration and invasion effects on skin cancer cells (Laomethakorn et al., 2023[84]); 9-O-phenylsulfonyl-berberines exhibit stronger lipid-lowering effects than native BBR (Kong et al., 2022[80]); and 9-N-n-alkyl BBR demonstrates greater glucose-lowering activity (Khvostov et al., 2022[79]). Additionally, dual substitution at C-9 and C-13, such as in 9-O-substituted-13-octylberberine, improves lipophilicity and oncogene inhibition, showing stronger anti-HCC activity via cell cycle arrest and mitochondrial apoptosis pathways (Chen et al., 2022[15]).

In addition to modifications at C-9 and C-13, BBR analogs such as 5d (with a hydroxyl group at C-11) and 7b (featuring an oxygen atom introduced into ring B and altered methoxy group positioning) enhance anti-breast cancer effects by inhibiting p300/CBP histone acetyltransferases (Zhong et al., 2023[267]; Lai et al., 2024[83]).

Beyond carbon modifications, BBR can also be conjugated with short- or medium-chain fatty acid salts (e.g., butyrate/decanoate) to enhance pro-apoptotic effects in melanoma (Xu et al., 2022[223]). Its conversion into organic acid salts (such as fumarate and succinate) improves glycemic control (Cui et al., 2018[27]), and co-administration of salified BBR with silybin enhances therapeutic efficacy against NAFLD (Ma et al., 2024[123]).

### Thermo-sensitive hydrogels

Thermo-sensitive hydrogels can undergo a sol-gel phase transition at near-physiological temperatures, making them effective drug carriers for improving targeting and minimizing systemic toxicity (Fan et al., 2022[36]; Chen et al., 2024[13]). When used as carriers for BBR or BBR combined with evodiamine, these in situ thermo-sensitive hydrogels administered intranasally have been shown to enhance brain targeting. This delivery system significantly alleviated depressive-like behaviors in animals subjected to chronic unpredictable mild stress (CUMS) by modulating monoamine neurotransmitter levels and mitochondrial function, outperforming conventional administration routes (Wang et al., 2020[192]; Xu et al., 2021[222]).

## Conclusion

BBR exhibits significant therapeutic potential across diverse pathologies including cancer, digestive disorders, metabolic syndrome, cardiovascular conditions, and neurological disorders, primarily through pleiotropic mechanisms such as metabolic reprogramming, immune regulation, cellular stress response coordination, gene expression, and gut microbiota modulation. 

In basic research, current evidence suggests that the systemic pharmacological actions of BBR are primarily mediated by its regulation of key receptors, enzymes, ncRNAs, and other functional molecules. However, its direct molecular targets remain unclear. Future studies should focus on identifying the core direct targets of BBR and elucidating the downstream signaling mechanisms involved, in order to better characterize its pharmacological effects across different disease models.

At the same time, BBR's poor oral bioavailability remains a major barrier to clinical translation. To address this issue, interdisciplinary collaboration among pharmacology, materials science, chemistry, and biomedical engineering is essential to develop more effective delivery systems such as nano formulations or controlled-release preparations. The design of structurally optimized BBR analogues may also improve its bioavailability and therapeutic potential.

In clinical research, although numerous preclinical studies have confirmed BBR's therapeutic potential in various diseases, high-quality randomized controlled trials (RCTs) remain relatively limited. Future efforts should focus on conducting more rigorous clinical studies to clarify its efficacy and safety. Moreover, given that BBR has demonstrated synergistic or protective effects when combined with other drugs in multiple studies, its role as a sensitizer or protective agent in combination therapies holds promise as a more practical application strategy.

Collectively, addressing these barriers through interdisciplinary collaboration will accelerate BBR's transition from traditional medicine to a broad-spectrum therapeutic agent in modern precision medicine.

## Notes

Jianyu Hao and Xinjuan Liu (Department of Gastroenterology, Beijing Chao-Yang Hospital, Capital Medical University, No.8, South Road of Workers Stadium, Chaoyang District, Beijing, 100020, China; E-mail: liuxinjuan@mail.ccmu.edu.cn) contributed equally as corresponding author.

## Declaration

### Conflict of interest 

The authors declare that they have no conflict of interest.

### Author contributions

Jianyu Hao conceived and designed the structure of the review. Haoxuan Cheng conducted the literature search and drafted the initial manuscript. Xinyu Li, Yanqi Wang, and Wanqing Deng prepared the table and figures. Guangyong Sun and Dong Zhang contributed to data interpretation and critically revised the manuscript for important intellectual content. Xinjuan Liu supervised the overall project and provided key revisions to the final manuscript.

### Declaration of Generative AI and AI-as- sisted technologies in the writing process

The authors used ChatGPT-assisted technologies only for grammar and language polishing.

### Statement of financial support

This work was supported by the Beijing Key Clinical Specialty Project.

### Data availability 

Data sharing is not applicable to this article as no datasets were generated or analyzed during the current study.

## Figures and Tables

**Table 1:**
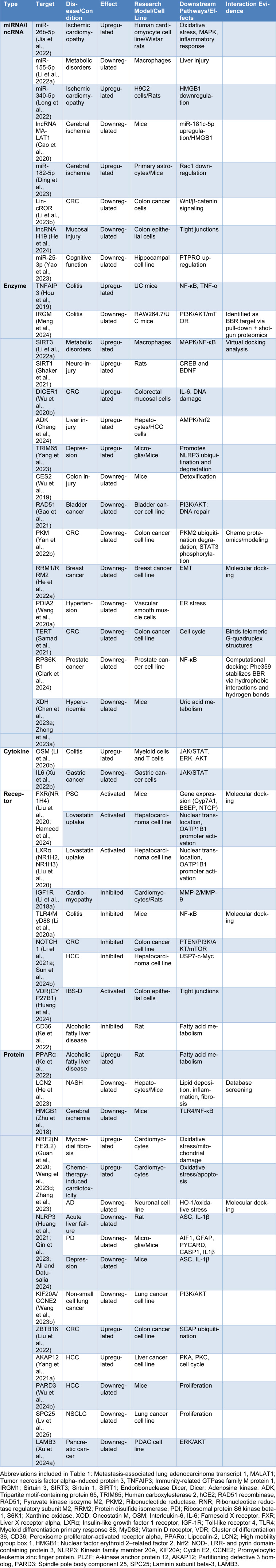
Potential targets of BBR

**Figure 1:**
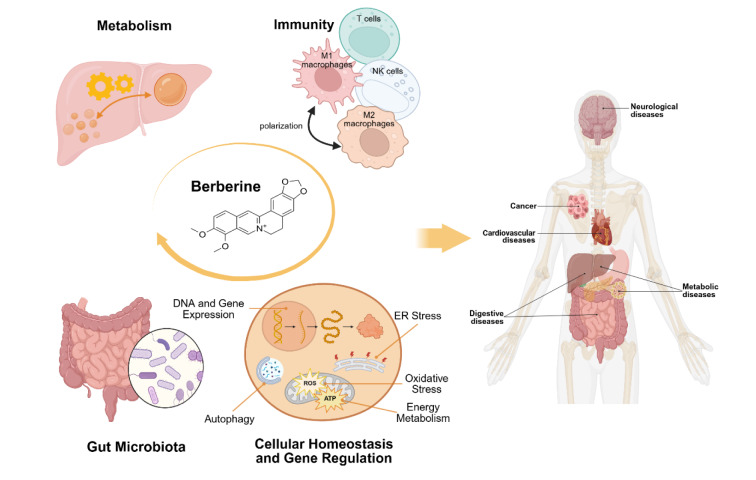
Graphical abstract

## References

[1] Abudureyimu M, Yu W, Cao RY, Zhang Y, Liu H, Zheng H (2020). Berberine promotes cardiac function by upregulating PINK1/parkin-mediated mitophagy in heart failure. Front Physiol.

[2] Aleissa MS, AL-Zharani M, Alneghery LM, Aleissa AM (2023). Berberine enhances the sensitivity of radiotherapy in ovarian cancer cell line (SKOV-3). Saudi Pharmaceutical Journal.

[3] Ali F, Alom S, Zaman MK (2021). Berberine: A comprehensive review on its isolation, biosynthesis, chemistry and pharmacology. Chem Asian J.

[4] Ali SA, Datusalia AK (2024). Berberine attenuates ECM accumulation and the progression of acute liver failure through inhibition of NLRP3 inflammasome signalling. Toxicology and Applied Pharmacology.

[5] Alnuqaydan AM, Almutary AG, Azam M, Manandhar B, Yin GHS, Yen LL (2022). Evaluation of the cytotoxic activity and anti-migratory effect of berberine–phytantriol liquid crystalline nanoparticle formulation on non-small-cell lung cancer In vitro. Pharmaceutics.

[6] Blais JE, Huang X, Zhao JV (2023). Overall and Sex-Specific Effect of Berberine for the Treatment of Dyslipidemia in Adults: A Systematic Review and Meta-Analysis of Randomized Placebo-Controlled Trials. Drugs.

[7] Blevins HM, Xu Y, Biby S, Zhang S (2022). The NLRP3 inflammasome pathway: A review of mechanisms and inhibitors for the treatment of inflammatory diseases. Front Aging Neurosci.

[8] Cao D, Liu M, Duan R, Tao Y, Zhou J, Fang W (2020). The lncRNA Malat1 functions as a ceRNA to contribute to berberine-mediated inhibition of HMGB1 by sponging miR-181c-5p in poststroke inflammation. Acta Pharmacol Sin.

[9] Chang W, Li K, Guan F, Yao F, Yu Y, Zhang M (2016). Berberine Pretreatment Confers Cardioprotection Against Ischemia-Reperfusion Injury in a Rat Model of Type 2 Diabetes. J Cardiovasc Pharmacol Ther.

[10] Chang W, Zhang M, Li J, Meng Z, Xiao D, Wei S (2012). Berberine attenuates ischemia-reperfusion injury via regulation of adenosine-5′-monophosphate kinase activity in both non-ischemic and ischemic areas of the rat heart. Cardiovasc Drugs Ther.

[11] Chen C, Lin Q, Zhu X-Y, Xia J, Mao T, Chi T (2021). Pre-clinical evidence: berberine as a promising cardioprotective candidate for myocardial ischemia/reperfusion injury, a systematic review, and meta-analysis. Front Cardiovasc Med.

[12] Chen C, Liu X, Deng B (2024). Protective Effects of Berberine on Nonalcoholic Fatty Liver Disease in db/db Mice via AMPK/SIRT1 Pathway Activation. Curr Med Sci.

[13] Chen H, Xu J, Sun J, Jiang Y, Zheng W, Hu W (2024). Recent advances on thermosensitive hydrogels-mediated precision therapy. Asian Journal of Pharmaceutical Sciences.

[14] Chen H, Zhang F, Zhang J, Zhang X, Guo Y, Yao Q (2020). A Holistic View of Berberine Inhibiting Intestinal Carcinogenesis in Conventional Mice Based on Microbiome-Metabolomics Analysis. Front Immunol.

[15] Chen J, Duan Y, Yu X, Zhong J, Bai J, Li NG, Zhu Z, Xu J (2022). Development of novel 9-O-substituted-13-octylberberine derivatives as potential anti-hepatocellular carcinoma agents. J Enzyme Inhib Med Chem.

[16] Chen M, Li L, Liu C, Song L (2020). Berberine attenuates Aβ-induced neuronal damage through regulating miR-188/NOS1 in Alzheimer’s disease. Mol Cell Biochem.

[17] Chen P, Dai C-H, Shi Z-H, Wang Y, Wu J-N, Chen K (2021). Synergistic inhibitory effect of berberine and icotinib on non-small cell lung cancer cells via inducing autophagic cell death and apoptosis. Apoptosis.

[18] Chen P, Li Y, Xiao L (2021). Berberine ameliorates nonalcoholic fatty liver disease by decreasing the liver lipid content via reversing the abnormal expression of MTTP and LDLR. Exp Ther Med.

[19] Chen Q, Li D, Wu F, He X, Zhou Y, Sun C (2023). Berberine Regulates the Metabolism of Uric Acid and Modulates IntestinalFlora in Hyperuricemia Rats Model. CCHTS.

[20] Chen S, Chen Z, Wang Y, Hao W, Yuan Q, Zhou H (2022). Targeted delivery of Chinese herb pair-based berberine/tannin acid self-assemblies for the treatment of ulcerative colitis. Journal of Advanced Research.

[21] Chen Y, Hao Z, Zhao H, Duan X, Jia D, Li K (2023). Berberine alleviates intestinal barrier dysfunction in glucolipid metabolism disorder hamsters by modulating gut microbiota and gut‐microbiota‐related tryptophan metabolites. J Sci Food Agric.

[22] Chen Y, Jiang Z, Xu J, Zhang J, Sun R, Zhou J (2021). Improving the ameliorative effects of berberine and curcumin combination via dextran-coated bilosomes on non-alcohol fatty liver disease in mice. J Nanobiotechnol.

[23] Chen Y, Li Q, Zhao S, Sun L, Yin Z, Wang X (2023). Berberine protects mice against type 2 diabetes by promoting PPARγ-FGF21-GLUT2-regulated insulin sensitivity and glucose/lipid homeostasis. Biochem Pharmacol.

[24] Cheng J, Yan G, Tan W, Qin Z, Xie Q, Liu Y (2024). Berberine alleviates fructose-induced hepatic injury via ADK/AMPK/Nrf2 pathway: A novel insight. Biomedicine & Pharmacotherapy.

[25] Clark A, Villarreal MR, Huang S-B, Jayamohan S, Rivas P, Hussain SS (2024). Targeting S6K/NFκB/ SQSTM1/Polθ signaling to suppress radiation resistance in prostate cancer. Cancer Lett.

[26] Cuan X, Yang X, Zhu W, Zhao Y, Luo R, Huang Y (2023). Antitumor effects of erlotinib in combination with berberine in A431 cells. BMC Pharmacol Toxicol.

[27] Cui H-X, Hu Y-N, Li J-W, Yuan K, Guo Y (2018). Preparation and evaluation of antidiabetic agents of berberine organic acid salts for enhancing the bioavailability. Molecules (Basel).

[28] Deng J, Zhao L, Yuan X, Li Y, Shi J, Zhang H (2022). Pre-Administration of Berberine Exerts Chemopreventive Effects in AOM/DSS-Induced Colitis-Associated Carcinogenesis Mice via Modulating Inflammation and Intestinal Microbiota. Nutrients.

[29] Dian L, Xu Z, Sun Y, Li J, Lu H, Zheng M (2022). Berberine alkaloids inhibit the proliferation and metastasis of breast carcinoma cells involving Wnt/β-catenin signaling and EMT. Phytochemistry.

[30] Ding W, Gu Q, Liu M, Zou J, Sun J, Zhu J (2023). Astrocytes-derived exosomes pre-treated by berberine inhibit neuroinflammation after stroke via miR-182-5p/Rac1 pathway. Int Immunopharmacol.

[31] Dong Y, Fan H, Zhang Z, Jiang F, Li M, Zhou H (2022). Berberine ameliorates DSS-induced intestinal mucosal barrier dysfunction through microbiota-dependence and Wnt/β-catenin pathway. Int J Biol Sci.

[32] Du J, Ding X, Zhang X, Zhao X, Shan H, Wang F (2018). Berberine attenuate staphylococcal enterotoxin B-mediated acute liver injury via regulating HDAC expression. AMB Expr.

[33] Du M, Liu X, Ji X, Wang Y, Liu X, Zhao C (2025). Berberine alleviates enterotoxigenic Escherichia coli-induced intestinal mucosal barrier function damage in a piglet model by modulation of the intestinal microbiome. Front Nutr.

[34] Du Y, Khan M, Fang N, Ma F, Du H, Tan Z (2022). Berberine attenuates cell motility via inhibiting inflammation-mediated lysyl hydroxylase-2 and glycolysis. Front Pharmacol.

[35] Duan H, Hu J, Deng Y, Zou J, Ding W, Peng Q (2023). Berberine mediates the production of butyrate to ameliorate cerebral ischemia via the gut microbiota in mice. Nutrients.

[36] Fan R, Cheng Y, Wang R, Zhang T, Zhang H, Li J (2022). Thermosensitive hydrogels and advances in their application in disease therapy. Polymers.

[37] Fang X, Wu H, Wang X, Lian F, Li M, Miao R (2022). Modulation of gut microbiota and metabolites by berberine in treating mice with disturbances in glucose and lipid metabolism. Front Pharmacol.

[38] Fang Y, Zhang J, Zhu S, He M, Ma S, Jia Q (2021). Berberine ameliorates ovariectomy-induced anxiety-like behaviors by enrichment in equol generating gut microbiota. Pharmacological Research.

[39] Fu Y, Wang Y, Wang X, Sun Y, Ren J, Fang B (2022). Responses of human gut microbiota abundance and amino acid metabolism in vitro to berberine. Food Funct.

[40] Gao X, Liu J, Fan D, Li X, Fang Z, Yan K (2021). Berberine enhances gemcitabine‑induced cytotoxicity in bladder cancer by downregulating Rad51 expression through inactivating the PI3K/akt pathway. Oncol Rep.

[41] Gao Y, Sun J, Wang S, Huxiao L, Xu Y, Zhang H (2023). DSPE-PEG polymer enhanced berberine absorption specifically in the small intestine of rats through paracellular passway. J Pharm Pharmacol.

[42] Gao Y, Wang F, Song Y, Liu H (2020). The status of and trends in the pharmacology of berberine: A bibliometric review [1985-2018]. Chin Med.

[43] Ge P, Qu S, Ni S, Yao Z, Qi Y, Zhao X (2023). Berberine ameliorates depression‐like behavior in CUMS mice by activating TPH1 and inhibiting IDO1‐associated with tryptophan metabolism. Phytother Res: Ptr.

[44] Ghobadi-Oghaz N, Asoodeh A, Mohammadi M (2022). Fabrication, characterization and in vitro cell exposure study of zein-chitosan nanoparticles for co-delivery of curcumin and berberine. Int J Biol Macromol.

[45] Gholampour F, Masoudi R, Khaledi M, Rooyeh MM, Farzad SH, Ataellahi F (2022). Berberis integerrima hydro-alcoholic root extract and its constituent berberine protect against cisplatin-induced nephro- and hepato-toxicity. Am J Med Sci.

[46] Gu W, Zhang M, Gao F, Niu Y, Sun L, Xia H (2022). Berberine regulates PADI4-related macrophage function to prevent lung cancer. Int Immunopharmacol.

[47] Guan X, Zheng X, Vong CT, Zhao J, Xiao J, Wang Y (2020). Combined effects of berberine and evodiamine on colorectal cancer cells and cardiomyocytes in vitro. Eur J Pharmacol.

[48] Guo Q, Wang C, Xue X, Hu B, Bao H (2021). SOCS1 mediates berberine-induced amelioration of microglial activated states in N9 microglia exposed to β amyloid. Biomed Res Int.

[49] Hajipour S, Farbood Y, Dianat M, Nesari A, Sarkaki A (2023). Effect of Berberine against Cognitive Deficits in Rat Model of Thioacetamide-Induced Liver Cirrhosis and Hepatic Encephalopathy (Behavioral, Biochemical, Molecular and Histological Evaluations). Brain Sciences.

[50] Hameed H, Irshad N, Yousaf MA, Mumtaz S, Sohail I (2024). Berberine ameliorates the progression of primary sclerosing cholangitis by activating farnesoid X receptor. Cell Biochem Biophys.

[51] Han C, Sun T, Xv G, Wang S, Gu J, Liu C (2019). Berberine ameliorates CCl4‑induced liver injury in rats through regulation of the Nrf2‑Keap1‑ARE and p53 signaling pathways. Mol Med Report.

[52] Harrison SA, Gunn N, Neff GW, Kohli A, Liu L, Flyer A (2021). A phase 2, proof of concept, randomised controlled trial of berberine ursodeoxycholate in patients with presumed non-alcoholic steatohepatitis and type 2 diabetes. Nat Commun.

[53] He H, Chai X, Li J, Li C, Wu X, Ye X (2023). LCN2 contributes to the improvement of nonalcoholic steatohepatitis by 8-Cetylberberine. Life Sciences.

[54] He J, Wei Q, Jiang R, Luan T, He S, Lu R (2022). The core-targeted RRM2 gene of berberine hydrochloride promotes breast cancer cell migration and invasion via the epithelial-mesenchymal transition. Pharmaceuticals (Basel).

[55] He Q, Chen B, Wang G, Zhou D, Zeng H, Li X (2022). Co-crystal of rosiglitazone with berberine ameliorates hyperglycemia and insulin resistance through the PI3K/AKT/TXNIP pathway In vivo and In vitro. Front Pharmacol.

[56] He R, Li Y, He Y, Wang Q, Zhang S, Chen S (2024). Berberine mitigates diclofenac-induced intestinal mucosal mechanical barrier dysfunction through the restoration of autophagy by inhibiting exosome-mediated lncRNA H19. Inflammopharmacology.

[57] Hosseini SH, Nazarian M, Rajabi S, Jafari-nozad AM, Mesbahzadeh B, Samargahndian S (2024). Protective effect of berberine nanoparticles against cardiotoxic effects of arsenic trioxide. Cardiovasc Toxicol.

[58] Hou Q, Zhu S, Zhang C, Huang Y, Guo Y, Li P (2019). Berberine improves intestinal epithelial tight junctions by upregulating A20 expression in IBS-D mice. Biomedicine & Pharmacotherapy.

[59] Hu F, Hu T, Qiao Y, Huang H, Zhang Z, Huang W (2024). Berberine inhibits excessive autophagy and protects myocardium against ischemia/reperfusion injury via the RhoE/AMPK pathway. Int J Mol Med.

[60] Hu J, Duan H, Zou J, Ding W, Wei Z, Peng Q (2024). METTL3-dependent N6-methyladenosine modification is involved in berberine-mediated neuroprotection in ischemic stroke by enhancing the stability of NEAT1 in astrocytes. Aging.

[61] Hu J, Shi Q, Xue C, Wang Q (2024). Berberine Protects against Hepatocellular Carcinoma Progression by Regulating Intrahepatic T Cell Heterogeneity. Advanced Science.

[62] Huang C, Liu H, Gong X-L, Wu L-Y, Wen B (2017). Effect of evodiamine and berberine on the interaction between DNMTs and target microRNAs during malignant transformation of the colon by TGF-β1. Oncol Rep.

[63] Huang C, Sun Y, Liao S, Chen Z, Lin H, Shen W (2022). Suppression of Berberine and Probiotics (in vitro and in vivo) on the Growth of Colon Cancer With Modulation of Gut Microbiota and Butyrate Production. Front Microbiol.

[64] Huang C, Tao L, Wang X-L, Pang Z (2019). Berberine reversed the epithelial-mesenchymal transition of normal colonic epithelial cells induced by SW480 cells through regulating the important components in the TGF-β pathway. J Cell Physiol.

[65] Huang M, He Y, Tian L, Yu L, Cheng Q, Li Z (2023). Gut microbiota-SCFAs-brain axis associated with the antidepressant activity of berberine in CUMS rats. J Affect Disord.

[66] Huang S, Liu H, Lin Y, Liu M, Li Y, Mao H (2021). Berberine protects against NLRP3 inflammasome via ameliorating autophagic impairment in MPTP-induced parkinson’s disease model. Front Pharmacol.

[67] Huang Y, Liu J, Chen G, Shen D, Zhu W, Chen X (2024). Berberine Enhances Intestinal Mucosal Barrier Function by Promoting Vitamin D Receptor Activity. Chin J Integr Med.

[68] Ibrahim D, Khater SI, Abdelfattah-Hassan A, Alqahtani LS, Metwally AS, Bazeed SM (2023). Prospects of new targeted nanotherapy combining liponiosomes with berberine to combat colorectal cancer development: An in vivo experimental model. Int J Pharm.

[69] Javed Iqbal M, Quispe C, Javed Z, Sadia H, Qadri QR, Raza S (2021). Nanotechnology-based strategies for berberine delivery system in cancer treatment: Pulling strings to keep berberine in power. Front Mol Biosci.

[70] Jia J, Zhang K, Zhou X, Ma J, Liu X, Xiang A (2019). Berberine-loaded solid proliposomes prepared using solution enhanced dispersion by supercritical CO2: Sustained release and bioavailability enhancement. J Drug Delivery Sci Technol.

[71] Jia X, Shao W, Tian S (2022). Berberine alleviates myocardial ischemia–reperfusion injury by inhibiting inflammatory response and oxidative stress: the key function of miR-26b-5p-mediated PTGS2/MAPK signal transduction. Pharm Biol.

[72] Jing W, Dong S, Luo X, Liu J, Wei B, Du W (2021). Berberine improves colitis by triggering AhR activation by microbial tryptophan catabolites. Pharmacological Research.

[73] Ju J, Li J, Lin Q, Xu H (2018). Efficacy and safety of berberine for dyslipidaemias: A systematic review and meta-analysis of randomized clinical trials. Phytomedicine.

[74] Kalaiarasi A, Anusha C, Sankar R, Rajasekaran S, John Marshal J, Muthusamy K (2016). Plant Isoquinoline Alkaloid Berberine Exhibits Chromatin Remodeling by Modulation of Histone Deacetylase To Induce Growth Arrest and Apoptosis in the A549 Cell Line. J Agric Food Chem.

[75] Kang Y-H, Wang J-H, Lee J-S, Hwang S-J, Lee N-H, Son C-G (2024). Berberine inhibits colorectal liver metastasis via modulation of TGF-β in a cecum transplant mouse model. Eur J Med Res.

[76] Ke X, Zhang R, Li P, Zuo L, Wang M, Yang J (2022). Hydrochloride Berberine ameliorates alcohol-induced liver injury by regulating inflammation and lipid metabolism. Biochemical and Biophysical Research Communications.

[77] Khaled AM, Othman MS, Obeidat ST, Aleid GM, Aboelnaga SM, Fehaid A (2024). Green-synthesized silver and selenium nanoparticles using berberine: a comparative assessment of In vitro anticancer potential on human hepatocellular carcinoma cell line (HepG2). Cells.

[78] Khater SI, Almanaa TN, Fattah DMA, Khamis T, Seif MM, Dahran N (2023). Liposome-Encapsulated Berberine Alleviates Liver Injury in Type 2 Diabetes via Promoting AMPK/mTOR-Mediated Autophagy and Reducing ER Stress: Morphometric and Immunohistochemical Scoring. Antioxidants (Basel).

[79] Khvostov MV, Gladkova ED, Borisov SA, Fedotova MS, Zhukova NA, Marenina MK (2022). 9-N-n-alkyl berberine derivatives: hypoglycemic activity evaluation. Pharmaceutics.

[80] Kong Y, Yi Y-J, Liu X-Q, Yu P, Zhao L-G, Li D-D (2022). Discovery and structural optimization of 9-O-phenylsulfonyl-berberines as new lipid-lowering agents. Bioorganic Chemistry.

[81] Koperska A, Moszak M, Seraszek-Jaros A, Bogdanski P, Szulinska M (2024). Does berberine impact anthropometric, hepatic, and metabolic parameters in patients with metabolic dysfunction-associated fatty liver disease? Randomized, double-blind placebo-controlled trial. J Physiol Pharmacol: Off J Pol Physiol Soc.

[82] Kowdley KV, Forman L, Eksteen B, Gunn N, Sundaram V, Landis C (2022). A randomized, dose-finding, proof-of-concept study of berberine ursodeoxycholate in patients with primary sclerosing cholangitis. Am J Gastroenterol.

[83] Lai R, Lin Z, Yang C, Hai L, Yang Z, Guo L (2024). Novel berberine derivatives as p300 histone acetyltransferase inhibitors in combination treatment for breast cancer. Eur J Med Chem.

[84] Laomethakorn P, Tayeh M, Samosorn S, Tananyuthawongse C, Watanapokasin R (2023). 13-butoxyberberine bromide inhibits migration and invasion in skin cancer A431 cells. Molecules.

[85] Li C, Ai G, Wang Y, Lu Q, Luo C, Tan L (2020). Oxyberberine, a novel gut microbiota-mediated metabolite of berberine, possesses superior anti-colitis effect: Impact on intestinal epithelial barrier, gut microbiota profile and TLR4-MyD88-NF-κB pathway. Pharmacological Research.

[86] Li D, Yang C, Zhu J, Lopez E, Zhang T, Tong Q (2022). Berberine remodels adipose tissue to attenuate metabolic disorders by activating sirtuin 3. Acta Pharmacol Sin.

[87] Li D, Zhang Y, Liu K, Zhao Y, Xu B, Xu L (2017). Berberine inhibits colitis-associated tumorigenesis via suppressing inflammatory responses and the consequent EGFR signaling-involved tumor cell growth. Lab Investig;J Tech Methods Pathol.

[88] Li G, Xing W, Zhang M, Geng F, Yang H, Zhang H (2018). Antifibrotic cardioprotection of berberine via downregulating myocardial IGF-1 receptor-regulated MMP-2/MMP-9 expression in diabetic rats. Am J Physiol Heart Circ Physiol.

[89] Li G, Zhang C, Liang W, Zhang Y, Shen Y, Tian X (2021). Berberine regulates the Notch1/PTEN/PI3K/AKT/ mTOR pathway and acts synergistically with 17-AAG and SAHA in SW480 colon cancer cells. Pharm Biol.

[90] Li H, Feng C, Fan C, Yang Y, Yang X, Lu H (2020). Intervention of oncostatin M-driven mucosal inflammation by berberine exerts therapeutic property in chronic ulcerative colitis. Cell Death Dis.

[91] Li H, Liu N-N, Li J-R, Dong B, Wang M-X, Tan J-L (2022). Combined Use of Bicyclol and Berberine Alleviates Mouse Nonalcoholic Fatty Liver Disease. Front Pharmacol.

[92] Li H, Yang W, Wu X, Tian L, Zhang W, Tian H (2024). Cationic fructan-based pH and intestinal flora dual stimulation nanoparticle with berberine for targeted therapy of IBD. International Journal of Biological Macromolecules.

[93] Li J, Dan W, Zhang C, Liu N, Wang Y, Liu J (2024). Exploration of Berberine Against Ulcerative Colitis via TLR4/NF-κB/HIF-1α Pathway by Bioinformatics and Experimental Validation. DDDT.

[94] Li J, Li J, Ni J, Zhang C, Jia J, Wu G (2022). Berberine Relieves Metabolic Syndrome in Mice by Inhibiting Liver Inflammation Caused by a High-Fat Diet and Potential Association With Gut Microbiota. Front Microbiol.

[95] Li J, Liu F, Jiang S, Liu J, Chen X, Zhang S (2018). Berberine hydrochloride inhibits cell proliferation and promotes apoptosis of non-small cell lung cancer via the suppression of the MMP2 and bcl-2/bax signaling pathways. Oncol Lett.

[96] Li J, Zhang S, Wu L, Pei M, Jiang Y (2021). Berberine inhibited metastasis through miR-145/MMP16 axis in vitro. J Ovarian Res.

[97] Li M, Dang Y, Li Q, Zhou W, Zuo J, Yao Z (2019). Berberine alleviates hyperglycemia by targeting hepatic glucokinase in diabetic db/db mice. Sci Rep.

[98] Li S, Cai X, Chen L, Lin M, Zhu Z, Xiao H (2023). Inhibition of hepatocellular carcinoma growth via modulation of the miR-221/SOX11 axis by curcumin and berberine. PeerJ.

[99] Li S, Shi C, Fu W, Zhang J (2023). Berberine inhibits tumour growth in vivo and in vitro through suppressing the lincROR-Wnt/β-catenin regulatory axis in colorectal cancer. J Pharm Pharmacol.

[100] Li X, Chen J, Feng W, Wang C, Chen M, Li Y (2023). Berberine ameliorates iron levels and ferroptosis in the brain of 3 × Tg-AD mice. Phytomedicine.

[101] Li X, Su Y, Li N, Zhang F-R, Zhang N (2021). Berberine attenuates MPP+-induced neuronal injury by regulating LINC00943/miR-142-5p/KPNA4/NF-κB pathway in SK-N-SH cells. Neurochem Res.

[102] Li X, Xu S, Zhang Y, Li K, Gao X-J, Guo M (2022). Berberine Depresses Inflammation and Adjusts Smooth Muscle to Ameliorate Ulcerative Colitis of Cats by Regulating Gut Microbiota. Microbiol Spectr.

[103] Li X-D, Wang Z, Wang X-R, Shao D, Zhang X, Li L (2019). Berberine-loaded Janus gold mesoporous silica nanocarriers for chemo/radio/photothermal therapy of liver cancer and radiation-induced injury inhibition. Int J Nanomedicine.

[104] Liang Y, Ye C, Chen Y, Chen Y, Diao S, Huang M (2021). Berberine improves behavioral and cognitive deficits in a mouse model of alzheimer’s disease via regulation of β-amyloid production and endoplasmic reticulum stress. ACS Chem Neurosci.

[105] Liao W, Zhang R, Chen G, Zhu X, Wu W, Chen Z (2024). Berberine synergises with ferroptosis inducer sensitizing NSCLC to ferroptosis in p53-dependent SLC7A11-GPX4 pathway. Biomedicine & Pharmacotherapy.

[106] Liao Y, Chen K, Dong X, Li W, Li G, Huang G (2018). Berberine inhibits cardiac remodeling of heart failure after myocardial infarction by reducing myocardial cell apoptosis in rats. Exp Ther Med.

[107] Liao Z, Xie Y, Zhou B, Zou B, Xiao D, Liu W (2020). Berberine ameliorates colonic damage accompanied with the modulation of dysfunctional bacteria and functions in ulcerative colitis rats. Appl Microbiol Biotechnol.

[108] Lin H-J, Ho J-H, Tsai L-C, Yang F-Y, Yang L-L, Kuo C-D (2020). Synthesis and In vitro photocytotoxicity of 9-/13-lipophilic substituted berberine derivatives as potential anticancer agents. Moleculs (Basel).

[109] Ling Q, Fang J, Zhai C, Huang W, Chen Y, Zhou T (2023). Berberine induces SOCS1 pathway to reprogram the M1 polarization of macrophages via miR-155-5p in colitis-associated colorectal cancer. Eur J Pharmacol.

[110] Liu M, Zhu D, Wen J, Ding W, Huang S, Xia C (2020). Berberine promotes OATP1B1 expression and rosuvastatin uptake by inducing nuclear translocation of FXR and LXRα. Front Pharmacol.

[111] Liu X, Liang Q, Wang Y, Xiong S, Yue R (2024). Advances in the pharmacological mechanisms of berberine in the treatment of fibrosis. Front Pharmacol.

[112] Liu Y, Fang X, Li Y, Bing L, Li Y, Fang J (2022). Berberine suppresses the migration and invasion of colon cancer cells by inhibition of lipogenesis through modulation of promyelocytic leukemia zinc finger-mediated sterol-regulatory element binding proteins cleavage-activating protein ubiquitination. J Pharm Pharmacol.

[113] Liu Y, Liu X, Hua W, Wei Q, Fang X, Zhao Z (2018). Berberine inhibits macrophage M1 polarization via AKT1/SOCS1/NF-κB signaling pathway to protect against DSS-induced colitis. International Immunopharmacology.

[114] Liu Y-L, Guo T, Zhang Y-J, Tang S-C, Zhao X-M, He H-Y (2024). Berberine alleviates ischemic brain injury by enhancing autophagic flux via facilitation of TFEB nuclear translocation. Am J Chin Med.

[115] Long T, Pan W, Li F, Sheikh SA, Xie Q, Zhang C (2022). Berberine up‐regulates miR‐340‐5p to protect myocardial ischaemia/reperfusion from HMGB1‐mediated inflammatory injury. Esc Heart Fail.

[116] Lu T, Wang Y, Liu F, Zhang L, Huang S, Zhou Y (2023). Synergistic Inhibitory Effect of Berberine and Low-Temperature Plasma on Non-Small-Cell Lung Cancer Cells via PI3K-AKT-Driven Signaling Axis. Molecules.

[117] Lu Z, Lu F, Wu L, He B, Chen Z, Yan M (2021). Berberine attenuates non-alcoholic steatohepatitis by regulating chemerin/CMKLR1 signalling pathway and Treg/ Th17 ratio. Naunyn-Schmiedeberg’s Arch Pharmacol.

[118] Luo Y, Tian G, Zhuang Z, Chen J, You N, Zhuo L (2019). Berberine prevents non-alcoholic steatohepatitis-derived hepatocellular carcinoma by inhibiting inflammation and angiogenesis in mice. Am J Transl Res.

[119] Luo Z, Li Z, Liang Z, Wang L, He G, Wang D (2022). Berberine increases stromal production of Wnt molecules and activates Lgr5+ stem cells to promote epithelial restitution in experimental colitis. BMC Biol.

[120] Lv M, Chen X, Yang Q, Huang C, Lv Y, Zhang T (2025). Berberine restrains non-small cell lung cancer cell growth, invasion and glycolysis via inactivating the SPC25/NUF2 pathway. Naunyn Schmiedebergs Arch Pharmacol.

[121] Lyu Y, Li D, Yuan X, Li Z, Zhang J, Ming X (2022). Effects of combination treatment with metformin and berberine on hypoglycemic activity and gut microbiota modulation in db/db mice. Phytomedicine.

[122] Ma W, Long J, Dong L, Zhang J, Wang A, Zhang Y (2024). Uncovering the key pharmacodynamic material basis and possible molecular mechanism of xiaoke formulation improve insulin resistant through a comprehensive investigation. J Ethnopharmacol.

[123] Ma X, Yu X, Li R, Cui J, Yu H, Ren L (2024). Berberine-silybin salt achieves improved anti-nonalcoholic fatty liver disease effect through regulating lipid metabolism. J Ethnopharmacol.

[124] Maiti P, Plemmons A, Dunbar GL (2019). Combination treatment of berberine and solid lipid curcumin particles increased cell death and inhibited PI3K/akt/mTOR pathway of human cultured glioblastoma cells more effectively than did individual treatments. PLOS One.

[125] Martins R, Pinto Rolo A, Soeiro Teodoro J, Furtado E, Caetano Oliveira R, Tralhão J (2018). Addition of Berberine to Preservation Solution in an Animal Model of Ex Vivo Liver Transplant Preserves Mitochondrial Function and Bioenergetics from the Damage Induced by Ischemia/Reperfusion. Int J Mol Sci.

[126] Mehboodi D, Shahedi A, Namavar MR, Yadegari M, Vakili M (2024). Effect of berberine on the hippocampal structure, biochemical factors, memory, and blood–brain barrier in rat model of transient global cerebral ischemia. Phytother Res.

[127] Meng G, Li P, Du X, Feng X, Qiu F (2024). Berberine alleviates ulcerative colitis by inhibiting inflammation through targeting IRGM1. Phytomedicine.

[128] Mianowska M, Zaremba-Czogalla M, Zygmunt A, Mahmud M, Süss R, Gubernator J (2023). Dual Role of Vitamin C-Encapsulated Liposomal Berberine in Effective Colon Anticancer Immunotherapy. Pharmaceuticals.

[129] Moreira ES, Ames-Sibin AP, Bonetti CI, Leal LE, Peralta RM, de Sá-Nakanishi AB (2022). The short-term effects of berberine in the liver: Narrow margins between benefits and toxicity. Toxicol Lett.

[130] Mori S, Fujiwara-Tani R, Gyoten M, Nukaga S, Sasaki R, Ikemoto A (2023). Berberine induces combined cell death in gastrointestinal cell lines. Int J Mol Sci.

[131] Morrison DJ, Preston T (2016). Formation of short chain fatty acids by the gut microbiota and their impact on human metabolism. Gut Microbes.

[132] Ni L, Li Z, Ren H, Kong L, Chen X, Xiong M (2022). Berberine inhibits non-small cell lung cancer cell growth through repressing DNA repair and replication rather than through apoptosis. Clin Exp Pharmacol Physiol.

[133] Ni S, Yao Z, Wei X, Heng X, Qu S, Zhao X (2022). Vagus nerve stimulated by microbiota‐derived hydrogen sulfide mediates the regulation of berberine on microglia in transient middle cerebral artery occlusion rats. Phytother Res: Ptr.

[134] Nie Q, Li M, Huang C, Yuan Y, Liang Q, Ma X (2024). The clinical efficacy and safety of berberine in the treatment of non-alcoholic fatty liver disease: a meta-analysis and systematic review. J Transl Med.

[135] Nie Q, Peng WW, Wang Y, Zhong L, Zhang X, Zeng L (2022). β-catenin correlates with the progression of colon cancers and berberine inhibits the proliferation of colon cancer cells by regulating the β-catenin signaling pathway. Gene.

[136] Noh J-W, Jun M-S, Yang H-K, Lee B-C (2022). Cellular and Molecular Mechanisms and Effects of Berberine on Obesity-Induced Inflammation. Biomedicines.

[137] Okuno K, Garg R, Yuan Y-C, Tokunaga M, Kinugasa Y, Goel A (2022). Berberine and oligomeric proanthocyanidins exhibit synergistic efficacy through regulation of PI3K-akt signaling pathway in colorectal cancer. Front Oncol.

[138] Othman MS, Obeidat ST, Al-Bagawi AH, Fareid MA, Fehaid A, Abdel Moneim AE (2022). Green-synthetized selenium nanoparticles using berberine as a promising anticancer agent. Journal of Integrative Medicine.

[139] Palma TV, Bianchin NB, de Oliveira JS, Assmann CE, das Neves Oliveira M, Schetinger MRC (2022). Berberine increases the expression of cytokines and proteins linked to apoptosis in human melanoma cells. Mol Biol Rep.

[140] Panigrahi A, Mohanty S (2023). Efficacy and safety of HIMABERB® Berberine on glycemic control in patients with prediabetes: double-blind, placebo-controlled, and randomized pilot trial. BMC Endocr Disord.

[141] Pu Z, Sun Y, Jiang H, Hou Q, Yan H, Wen H (2021). Effects of Berberine on Gut Microbiota in Patients with Mild Metabolic Disorders Induced by Olanzapine. Am J Chin Med.

[142] Qi Y, Liu G (2021). Berberine-10-hydroxy camptothecine-loaded lipid microsphere for the synergistic treatment of liver cancer by inhibiting topoisomerase and HIF-1α. Drug Delivery.

[143] Qian K, Tang C-Y, Chen L-Y, Zheng S, Zhao Y, Ma L-S (2021). Berberine reverses breast cancer multidrug resistance based on fluorescence pharmacokinetics In vitro and In vivo. ACS Omega.

[144] Qian Y, Kang Z, Zhao L, Chen H, Zhou C, Gao Q (2023). Berberine might block colorectal carcinogenesis by inhibiting the regulation of B-cell function by Veillonella parvula. Chin Med J (Engl).

[145] Qin X, Jiang M, Zhao Y, Gong J, Su H, Yuan F (2020). Berberine protects against diabetic kidney disease via promoting PGC‐1α‐regulated mitochondrial energy homeostasis. Br J Pharmacol.

[146] Qin Z, Shi D-D, Li W, Cheng D, Zhang Y-D, Zhang S (2023). Berberine ameliorates depression-like behaviors in mice via inhibiting NLRP3 inflammasome-mediated neuroinflammation and preventing neuroplasticity disruption. J Neuroinflammation.

[147] Qin-Wei Z, Yong-Guang L (2016). Berberine attenuates myocardial ischemia reperfusion injury by suppressing the activation of PI3K/AKT signaling. Exp Ther Med.

[148] Qiu D, Zhang W, Song Z, Xue M, Zhang Y, Yang Y (2022). Berberine suppresses cecal ligation and puncture induced intestinal injury by enhancing treg cell function. Int Immunopharmacol.

[149] Rahman Z, Shaikh AS, Rao KV, Dandekar MP (2024). Oxyberberine protects middle cerebral artery occlusion triggered cerebral injury through TLR4/NLRP3 pathway in rats. J Chem Neuroanat.

[150] Raju M, Kunde SS, Auti ST, Kulkarni YA, Wairkar S (2021). Berberine loaded nanostructured lipid carrier for alzheimer’s disease: design, statistical optimization and enhanced in vivo performance. Life Sciences.

[151] Ren G, Guo J-H, Qian Y-Z, Kong W-J, Jiang J-D (2020). Berberine improves glucose and lipid metabolism in HepG2 cells through AMPKα1 activation. Front Pharmacol.

[152] Rezaeian L, Kalalian-Moghaddam H, Mohseni F, Khaksari M, Rafaiee R (2020). Effects of berberine hydrochloride on methamphetamine-induced anxiety behaviors and relapse in rats. Iran J Basic Med Sci.

[153] Saleh SR, Abd-Elmegied A, Aly Madhy S, Khattab SN, Sheta E, Elnozahy FY (2024). Brain-targeted tet-1 peptide-PLGA nanoparticles for berberine delivery against STZ-induced alzheimer’s disease in a rat model: alleviation of hippocampal synaptic dysfunction, tau pathology, and amyloidogenesis. Int J Pharm.

[154] Samad MA, Saiman MZ, Abdul Majid N, Karsani SA, Yaacob JS (2021). Berberine Inhibits Telomerase Activity and Induces Cell Cycle Arrest and Telomere Erosion in Colorectal Cancer Cell Line, HCT 116. Molecules (Basel).

[155] Shah D, Challagundla N, Dave V, Patidar A, Saha B, Nivsarkar M (2022). Berberine mediates tumor cell death by skewing tumor-associated immunosuppressive macrophages to inflammatory macrophages. Phytomedicine.

[156] Shaker FH, El-Derany MO, Wahdan SA, El-Demerdash E, El-Mesallamy HO (2021). Berberine ameliorates doxorubicin-induced cognitive impairment (chemobrain) in rats. Life Sci.

[157] Shi L, Wang W, Jing C, Hu J, Liao X (2025). Berberine and health outcomes: An overview of systematic reviews. BMC Complementary Med Ther.

[158] Shi Y, Hu J, Geng J, Hu T, Wang B, Yan W (2018). Berberine treatment reduces atherosclerosis by mediating gut microbiota in apoE-/- mice. Biomedicine & Pharmacotherapy.

[159] Shou J-W, Shaw P-C (2023). Berberine activates PPARδ and promotes gut microbiota-derived butyric acid to suppress hepatocellular carcinoma. Phytomedicine.

[160] Shu X, Li M, Cao Y, Li C, Zhou W, Ji G (2021). Berberine Alleviates Non-alcoholic Steatohepatitis Through Modulating Gut Microbiota Mediated Intestinal FXR Activation. Front Pharmacol.

[161] Singh IP, Mahajan S (2013). Berberine and its derivatives: a patent review (2009 - 2012). Expert Opin Ther Pat.

[162] Singh N, Sharma B (2018). Toxicological effects of berberine and sanguinarine. Front Mol Biosci.

[163] Song D, Hao J, Fan D (2020). Biological properties and clinical applications of berberine. Front Med.

[164] Suadoni MT, Atherton I (2021). Berberine for the treatment of hypertension: a systematic review. Complement Ther Clin Pract.

[165] Sun C, Dong S, Chen W, Li J, Luo E, Ji J (2024). Berberine alleviates alzheimer’s disease by regulating the gut microenvironment, restoring the gut barrier and brain-gut axis balance. Phytomedicine.

[166] Sun L, He M, Li F, Wu D, Zheng P, Zhang C (2024). Oxyberberine sensitizes liver cancer cells to sorafenib via inhibiting NOTCH1-USP7-c-Myc pathway. Hepatol Commun.

[167] Sun Q, Tao Q, Ming T, Tang S, Zhao H, Liu M (2023). Berberine is a suppressor of Hedgehog signaling cascade in colorectal cancer. Phytomedicine.

[168] Sun Q, Yang H, Liu M, Ren S, Zhao H, Ming T (2022). Berberine suppresses colorectal cancer by regulation of Hedgehog signaling pathway activity and gut microbiota. Phytomedicine.

[169] Sun R, Kong B, Yang N, Cao B, Feng D, Yu X (2021). The Hypoglycemic Effect of Berberine and Berberrubine Involves Modulation of Intestinal Farnesoid X Receptor Signaling Pathway and Inhibition of Hepatic Gluconeogenesis. Drug Metab Dispos: Biol Fate Chem.

[170] Sun X, Zhang Y, Cheng G, Zhu T, Zhang Z, Xiong L (2023). Berberine improves DSS-induced colitis in mice by modulating the fecal-bacteria-related bile acid metabolism. Biomedicine & Pharmacotherapy.

[171] Sun Y, Huang H, Zhan Z, Gao H, Zhang C, Lai J (2022). Berberine inhibits glioma cell migration and invasion by suppressing TGF-β1/COL11A1 pathway. Biochem Biophys Res Commun.

[172] Sun Y, Xia M, Yan H, Han Y, Zhang F, Hu Z (2018). Berberine attenuates hepatic steatosis and enhances energy expenditure in mice by inducing autophagy and fibroblast growth factor 21. Br J Pharmacol.

[173] Tai C-J, Jassey A, Liu C-H, Tai C-J, Richardson CD, Wong SH (2020). Targeting autophagy augments BBR-mediated cell death in human hepatoma cells harboring hepatitis C virus RNA. Cells.

[174] Tan Y, Li C, Zhou J, Deng F, Liu Y (2023). Berberine attenuates liver fibrosis by autophagy inhibition triggering apoptosis via the miR-30a-5p/ATG5 axis. Experimental Cell Research.

[175] Tang Y, Gao Y, Nie K, Wang H, Chen S, Su H (2024). Jiao-tai-wan and its effective component-berberine improve diabetes and depressive disorder through the cAMP/PKA/CREB signaling pathway. J Ethnopharmacol.

[176] Tang Y, Su H, Nie K, Wang H, Gao Y, Chen S (2024). Berberine exerts antidepressant effects in vivo and in vitro through the PI3K/AKT/CREB/BDNF signaling pathway. Biomed Pharmacother.

[177] Tong Y, Liu L, Wang R, Yang T, Wen J, Wei S (2021). Berberine Attenuates Chronic Atrophic Gastritis Induced by MNNG and Its Potential Mechanism. Front Pharmacol.

[178] Tu S, Huang Y, Tian H, Xu L, Wang X, Huang L (2024). Berberine enhances the function of intestinal stem cells in healthy and radiation-injured mice. International Immunopharmacology.

[179] Uma Maheswari RT, Ajithkumar V, Varalakshmi P, Rajan M (2023). CD44 tagged hyaluronic acid - chitosan liposome carrier for the delivery of berberine and doxorubicin into lung cancer cells. Int J Biol Macromol.

[180] Vishnoi K, Ke R, Saini KS, Viswakarma N, Nair RS, Das S (2021). Berberine represses β-catenin translation involving 4E-BPs in hepatocellular carcinoma cells. Mol Pharmacol.

[181] Wang C, Zou Q, Pu Y, Cai Z, Tang Y (2023). Berberine Rescues D-Ribose-Induced Alzheimer‘s Pathology via Promoting Mitophagy. IJMS.

[182] Wang D, Ren Y, Sun W, Gong J, Zou X, Dong H (2022). Berberine ameliorates glucose metabolism in diabetic rats through the alpha7 nicotinic acetylcholine receptor-related cholinergic anti-inflammatory pathway. Planta Med.

[183] Wang H, Chen S, Tang Y, Nie K, Gao Y, Wang Z (2024). Berberine promotes lacteal junction zippering and ameliorates diet-induced obesity through the RhoA/ ROCK signaling pathway. Phytomedicine.

[184] Wang J, Wei L-R, Liu Y-L, Ding C-Z, Guo F, Wang J (2021). Berberine activates the β-catenin/TCF4 signaling pathway by down-regulating miR-106b to promote GLP-1 production by intestinal L cells. European Journal of Pharmacology.

[185] Wang K, Gu C, Yu G, Lin J, Wang Z, Lu Q (2022). Berberine enhances the anti-hepatocellular carcinoma effect of NK92-MI cells through inhibiting IFN-gamma-mediated PD-L1 expression. Liver Res.

[186] Wang L, Deng L, Lin N, Shi Y, Chen J, Zhou Y (2020). Berberine inhibits proliferation and apoptosis of vascular smooth muscle cells induced by mechanical stretch via the PDI/ERS and MAPK pathways. Life Sci.

[187] Wang L, Zhou B-Q, Li Y-H, Jiang Q-Q, Cong W-H, Chen K-J (2022). Lactoferrin modification of berberine nanoliposomes enhances the neuroprotective effects in a mouse model of alzheimer’s disease. Neural Regener Res.

[188] Wang M, Ma Y, Yu G, Zeng B, Yang W, Huang C (2024). Integration of microbiome, metabolomics and transcriptome for in-depth understanding of berberine attenuates AOM/DSS-induced colitis-associated colorectal cancer. Biomedicine & Pharmacotherapy.

[189] Wang P, Li R, Li Y, Tan S, Jiang J, Liu H (2022). Berberine alleviates non-alcoholic hepatic steatosis partially by promoting SIRT1 deacetylation of CPT1A in mice. Gastroenterol Rep.

[190] Wang Q, Shen W, Shao W, Hu H (2024). Berberine alleviates cholesterol and bile acid metabolism disorders induced by high cholesterol diet in mice. Biochemical and Biophysical Research Communications.

[191] Wang Q, Wu H, Wu Q, Zhong S (2023). Berberine targets KIF20A and CCNE2 to inhibit the progression of nonsmall cell lung cancer via the PI3K/AKT pathway. Drug Dev Res.

[192] Wang Q-S, Li K, Gao L-N, Zhang Y, Lin K-M, Cui Y-L (2020). Intranasal delivery of berberine via in situ thermoresponsive hydrogels with non-invasive therapy exhibits better antidepressant-like effects. Biomater Sci.

[193] Wang T-T, Yu L-L, Zheng J-M, Han X-Y, Jin B-Y, Hua C-J (2024). Berberine inhibits ferroptosis and stabilizes atherosclerotic plaque through NRF2/SLC7A11/ GPX4 pathway. Chin J Integr Med.

[194] Wang X, Peng A, Huang C (2024). Suppression of colon cancer growth by berberine mediated by the intestinal microbiota and the suppression of DNA methyltransferases (DNMTs). Mol Cell Biochem.

[195] Wang X, Zhang J, Wang S, Song Z, Sun H, Wu F (2023). Berberine modulates gut microbiota to attenuate cerebral ferroptosis induced by ischemia-reperfusion in mice. European Journal of Pharmacology.

[196] Wang Y, Liao J, Luo Y, Li M, Su X, Yu B (2023). Berberine Alleviates Doxorubicin-Induced Myocardial Injury and Fibrosis by Eliminating Oxidative Stress and Mitochondrial Damage via Promoting Nrf-2 Pathway Activation. Int J Mol Sci.

[197] Wang Y, Liu H, Zheng M, Yang Y, Ren H, Kong Y (2021). Berberine Slows the Progression of Prediabetes to Diabetes in Zucker Diabetic Fatty Rats by Enhancing Intestinal Secretion of Glucagon-Like Peptide-2 and Improving the Gut Microbiota. Front Endocrinol.

[198] Wang Y, Tai Y-L, Zhao D, Zhang Y, Yan J, Kakiyama G (2021). Berberine Prevents Disease Progression of Nonalcoholic Steatohepatitis through Modulating Multiple Pathways. Cells.

[199] Wang Y, Tong Q, Ma S-R, Zhao Z-X, Pan L-B, Cong L (2021). Oral berberine improves brain dopa/dopamine levels to ameliorate parkinson’s disease by regulating gut microbiota. Signal Transduct Target Ther.

[200] Wang Y, Zhao D, Su L, Tai Y-L, Way GW, Zeng J (2024). Therapeutic potential of berberine in attenuating cholestatic liver injury: insights from a PSC mouse model. Cell Biosci.

[201] Wang Y, Zhou L, Li Y, Guo L, Zhou Z, Xie H (2017). The Effects of Berberine on Concanavalin A-Induced Autoimmune Hepatitis (AIH) in Mice and the Adenosine 5’-Monophosphate (AMP)-Activated Protein Kinase (AMPK) Pathway. Med Sci Monit.

[202] Wang Y, Zhou X, Zhao D, Wang X, Gurley EC, Liu R (2020). Berberine inhibits free fatty acid and LPS-induced inflammation via modulating ER stress response in macrophages and hepatocytes. PLoS One.

[203] Wang Z, Shao Y, Wu F, Luo D, He G, Liang J (2024). Berberine ameliorates vascular dysfunction by downregulating TMAO-endoplasmic reticulum stress pathway via gut microbiota in hypertension. Microbiol Res.

[204] Wang Z, Zhong Y, Xin M, Zhang J, Dong X, Zhang W (2024). Swiprosin-1 participates in the berberine-regulated AMPK/MLCK pathway to attenuate colitis-induced tight junction damage. Phytomedicine.

[205] Wei W, Yao J, Zhang T, Wen J, Zhang Z, Luo Y (2023). Network pharmacology reveals that berberine may function against alzheimer’s disease via the AKT signaling pathway. Front Neurosci.

[206] Wen J, Zhang Y-Q, Liu D-Q, Yao X-T, Jiang H, Zhang Y-B (2022). Demethylenetetrahydroberberine protects dopaminergic neurons in a mouse model of parkinson’s disease. Chin J Nat Med.

[207] Wu C, Liu Y, Liu W, Zou T, Lu S, Zhu C (2023). NNMT‐DNMT1 Axis is Essential for Maintaining Cancer Cell Sensitivity to Oxidative Phosphorylation Inhibition. Advanced Science.

[208] Wu J, Li Y, Sun S, Li W, Sun J, Zhu L (2024). The pH-sensitive chondroitin sulphate-based nanoparticles for co-delivery of doxorubicin and berberine enhance the treatment of breast cancer. Int J Biol Macromol.

[209] Wu J, Tan H-Y, Chan Y-T, Lu Y, Feng Z, Yuan H (2024). PARD3 drives tumorigenesis through activating Sonic Hedgehog signalling in tumour-initiating cells in liver cancer. J Exp Clin Cancer Res.

[210] Wu L, Meng X, Xu T, Zhang X, Zhou Y, Tong Z (2024). Berberine attenuates cognitive dysfunction and hippocampal apoptosis in rats with prediabetes. Chem Biol Drug Des.

[211] Wu M, Yang S, Wang S, Cao Y, Zhao R, Li X (2020). Effect of Berberine on Atherosclerosis and Gut Microbiota Modulation and Their Correlation in High-Fat Diet-Fed ApoE−/− Mice. Front Pharmacol.

[212] Wu W, Sun Y, Niu S, Li X, Chen L, Xie S (2025). Integrated Microbiome and Metabolomic to Explore the Mechanism of Coptisine in Alleviating Ulcerative Colitis. Phytother res: PTR.

[213] Wu X, Chen X, Liu H, He Z-W, Wang Z, Wei L-J (2020). Rescuing Dicer expression in inflamed colon tissues alleviates colitis and prevents colitis-associated tumorigenesis. Theranostics.

[214] Wu X, Li X, Dang Z, Jia Y (2018). Berberine demonstrates anti-inflammatory properties in helicobacter pylori-infected mice with chronic gastritis by attenuating the Th17 response triggered by the B cell-activating factor. J Cell Biochem.

[215] Wu Y, Jia Q, Tang Q, Deng H, He Y, Tang F (2024). Berberine-mediated Ferroptosis through System Xc-/GSH/ GPX4 Axis Inhibits Metastasis of Nasopharyngeal Carcinoma. J Cancer.

[216] Wu Y, Wang D, Yang X, Fu C, Zou L, Zhang J (2019). Traditional chinese medicine gegen qinlian decoction ameliorates irinotecan chemotherapy-induced gut toxicity in mice. Biomed Pharmacother = Biomed Pharmacother.

[217] Xia Q, Wu F, Wu W, Dong H, Huang Z, Xu L (2022). Berberine reduces hepatic ceramide levels to improve insulin resistance in HFD-fed mice by inhibiting HIF-2α. Biomedicine & Pharmacotherapy.

[218] Xia Y, Chen S, Cui J, Wang Y, Liu X, Shen Y (2021). Berberine suppresses bladder cancer cell proliferation by inhibiting JAK1-STAT3 signaling via upregulation of miR-17-5p. Biochem Pharmacol.

[219] Xie P, Ren Z, Lv J, Hu Y, Guan Z, Yu W (2020). Berberine Ameliorates Oxygen-glucose Deprivation/Reperfusion-induced Apoptosis by Inhibiting Endoplasmic Reticulum Stress and Autophagy in PC12 Cells. Curr Med Sci.

[220] Xu C, Pascual-Sabater S, Fillat C, Goel A (2024). The LAMB3-EGFR signaling pathway mediates synergistic anti-cancer effects of berberine and emodin in pancreatic cancer. Biochemical Pharmacology.

[221] Xu C, Zhao L, Zhou W, Li Y, Hu H, Wang Z (2023). Synergistic effect of berberine hydrochloride and dehydrocostus lactone in the treatment of ulcerative colitis: Take gut microbiota as the target. International Immunopharmacology.

[222] Xu D, Qiu C, Wang Y, Qiao T, Cui Y-L (2021). Intranasal co-delivery of berberine and evodiamine by self-assembled thermosensitive in-situ hydrogels for improving depressive disorder. Int J Pharm.

[223] Xu F, Liu M, Liao Y, Zhou Y, Zhang P, Zeng Y (2022). Improvement of anticancer effect of berberine by salt formation modifications. Phytomedicine.

[224] Xu M, Ren L, Fan J, Huang L, Zhou L, Li X (2022). Berberine inhibits gastric cancer development and progression by regulating the JAK2/STAT3 pathway and downregulating IL-6. Life Sci.

[225] Xu X, Li W, Yu Z, Zhang L, Duo T, Zhao Y (2022). Berberine Ameliorates Dextran Sulfate Sodium-Induced Ulcerative Colitis and Inhibits the Secretion of Gut Lysozyme via Promoting Autophagy. Metabolites.

[226] Xu X, Wu Y, Zhao Y, Liu A, Yi C, Zhang A (2024). Inhibition of Macrophage Pyroptosis─A New Therapeutic Strategy to Alleviate T-2 Toxin-Induced Subacute Liver Injury by Directly Competing with the Key Target. J Agric Food Chem.

[227] Xu X, Zhang L, Zhao Y, Xu B, Qin W, Yan Y (2020). Anti‑inflammatory mechanism of berberine on lipopolysaccharide‑induced IEC‑18 models based on comparative transcriptomics. Mol Med Rep.

[228] Xuan W, Wang H, Zhou P, Ye T, Gao H, Ye S (2020). Berberine ameliorates rats model of combined Alzheimer’s disease and type 2 diabetes mellitus via the suppression of endoplasmic reticulum stress. 3 Biotech.

[229] Yan J, Fang C, Yang G, Li J, Liu Y, Zhang L (2023). Identification of FtfL as a novel target of berberine in intestinal bacteria. BMC Biol.

[230] Yan S, Chang J, Hao X, Liu J, Tan X, Geng Z (2022). Berberine regulates short-chain fatty acid metabolism and alleviates the colitis-associated colorectal tumorigenesis through remodeling intestinal flora. Phytomedicine.

[231] Yan S-H, Hu L-M, Hao X-H, Liu J, Tan X-Y, Geng Z-R (2022). Chemoproteomics reveals berberine directly binds to PKM2 to inhibit the progression of colorectal cancer. Iscience.

[232] Yan X, Yuan C, Wang Z, Xu Z, Wu Z, Wang M (2024). Berberine modulates ovarian cancer autophagy and glycolysis through the LINC01123/P65/MAPK10 signaling axis. Phytomedicine.

[233] Yan Y, Li L, Wu K, Zhang G, Peng L, Liang Y (2022). A Combination of Baicalin and Berberine Hydrochloride Ameliorates Dextran Sulfate Sodium-Induced Colitis by Modulating Colon Gut Microbiota. Journal of Medicinal Food.

[234] Yang H, Cao G, Li X, Zhao Z, Wang Y, Xu F (2025). Berberine intervention mitigates myocardial ischemia-reperfusion injury in a rat model: mechanistic insights via miR-184 signaling. Biol: Targets Ther.

[235] Yang J, Yan H, Li S, Zhang M (2018). Berberine ameliorates MCAO induced cerebral ischemia/reperfusion injury via activation of the BDNF–TrkB–PI3K/akt signaling pathway. Neurochem Res.

[236] Yang L, Cao J, Wei J, Deng J, Hou X, Hao E (2021). Antiproliferative activity of berberine in HepG2 cells via inducing apoptosis and arresting cell cycle. Food Funct.

[237] Yang L, Huang Y, Chen F, Wang Y, Su K, Zhao M (2023). Berberine attenuates depression-like behavior by modulating the hippocampal NLRP3 ubiquitination signaling pathway through Trim65. Int Immunopharmacol.

[238] Yang L, Yu S, Yang Y, Wu H, Zhang X, Lei Y (2022). Berberine improves liver injury induced glucose and lipid metabolic disorders via alleviating ER stress of hepatocytes and modulating gut microbiota in mice. Bioorg Med Chem.

[239] Yang M, Yang C, Zhang Y, Yan X, Ma Y, Zhang Y (2022). An oral pH-activated “nano-bomb” carrier combined with berberine by regulating gene silencing and gut microbiota for site-specific treatment of ulcerative colitis. Biomater Sci.

[240] Yang S, Cao S, Li C, Zhang J, Liu C, Qiu F (2022). Berberrubine, a Main Metabolite of Berberine, Alleviates Non-Alcoholic Fatty Liver Disease via Modulating Glucose and Lipid Metabolism and Restoring Gut Microbiota. Front Pharmacol.

[241] Yang S, Cao S-J, Li C-Y, Zhang Q, Zhang B-L, Qiu F (2024). Berberine directly targets AKR1B10 protein to modulate lipid and glucose metabolism disorders in NAFLD. Journal of Ethnopharmacology.

[242] Yang T, Wang R, Liu H, Wang L, Li J, Wu S (2021). Berberine regulates macrophage polarization through IL-4-STAT6 signaling pathway in helicobacter pylori-induced chronic atrophic gastritis. Life Sci.

[243] Yang Y, Wu J, Jia L, Feng S, Qi Z, Yu H (2024). Berberine modulates microglial polarization by activating TYROBP in alzheimer’s disease. Phytomedicine.

[244] Yang Y-N, Wang Q-C, Xu W, Yu J, Zhang H, Wu C (2022). The berberine-enriched gut commensal Blautia producta ameliorates high-fat diet (HFD)-induced hyperlipidemia and stimulates liver LDLR expression. Biomedicine & Pharmacotherapy.

[245] Yao Z, Dong H, Zhu J, Du L, Luo Y, Liu Q (2023). Age-related decline in hippocampal tyrosine phosphatase PTPRO is a mechanistic factor in chemotherapy-related cognitive impairment. Jci Insight.

[246] Yarmohammadi F, Hayes AW, Karimi G (2022). The therapeutic effects of berberine against different diseases: A review on the involvement of the endoplasmic reticulum stress. Phytother Res: PTR.

[247] Ye Y, Liu X, Wu N, Han Y, Wang J, Yu Y (2021). Efficacy and safety of berberine alone for several metabolic disorders: A systematic review and meta-analysis of randomized clinical trials. Front Pharmacol.

[248] Yi J, Wu S, Tan S, Qin Y, Wang X, Jiang J (2021). Berberine alleviates liver fibrosis through inducing ferrous redox to activate ROS-mediated hepatic stellate cells ferroptosis. Cell Death Discov.

[249] Yi L, Zhu J, Dong S, Chen M, Li C (2020). Berberine exerts antidepressant-like effects via regulating miR-34a-synaptotagmin1/bcl-2 axis. Chin Herb Med.

[250] Yu H, Zhang S, Li R, Ma C, Zhang Q, Xia F (2024). Berberine alleviates inflammation and suppresses PLA2-COX-2-PGE2-EP2 pathway through targeting gut microbiota in DSS-induced ulcerative colitis. Biochemical and Biophysical Research Communications.

[251] Yu J, Zheng Y, Liu C, Xie Z, Liu Q, Yang S (2024). Multi-omics reveals the alleviating effect of berberine on ulcerative colitis through modulating the gut microbiome and bile acid metabolism in the gut-liver axis. Front Pharmacol.

[252] Yu M, Alimujiang M, Hu L, Liu F, Bao Y, Yin J (2021). Berberine alleviates lipid metabolism disorders via inhibition of mitochondrial complex I in gut and liver. Int J Biol Sci.

[253] Yue B, Gao R, Lv C, Yu Z, Wang H, Geng X (2021). Berberine improves irinotecan-induced intestinal mucositis without impairing the anti-colorectal cancer efficacy of irinotecan by inhibiting bacterial β-glucuronidase. Front Pharmacol.

[254] Zhai F, Wang J, Wan X, Liu Y, Mao X (2024). Dual anti-inflammatory effects of curcumin and berberine on acetaminophen-induced liver injury in mice by inhibiting NF-κB activation via PI3K/AKT and PPARγ signaling pathways. Biochemical and Biophysical Research Communications.

[255] Zhan Y, Han J, Xia J, Wang X (2021). Berberine suppresses mice depression behaviors and promotes hippocampal neurons growth through regulating the miR-34b-5p/miR-470-5p/BDNF axis. Neuropsychiatr Dis Treat.

[256] Zhang D, Ke L, Ni Z, Chen Y, Zhang L-H, Zhu S-H (2017). Berberine containing quadruple therapy for initial helicobacter pylori eradication. Medicine (Baltimore).

[257] Zhang M, Yang H, Yang E, Li J, Dong L (2021). Berberine decreases intestinal GLUT2 translocation and reduces intestinal glucose absorption in mice. Int J Mol Sci.

[258] Zhang N, Sheng M, Wu M, Zhang X, Ding Y, Lin Y (2019). Berberine protects steatotic donor undergoing liver transplantation via inhibiting endoplasmic reticulum stress-mediated reticulophagy. Exp Biol Med (Maywood).

[259] Zhang R, Lei B, Wu G, Wang Y, Huang Q (2023). Protective effects of berberine against β-amyloid-induced neurotoxicity in HT22 cells via the Nrf2/HO‐1 pathway. Bioorg Chem.

[260] Zhao L, Li H, Gao Q, Xu J, Zhu Y, Zhai M (2021). Berberine attenuates cerebral ischemia-reperfusion injury induced neuronal apoptosis by down-regulating the CNPY2 signaling pathway. Front Pharmacol.

[261] Zhao Y, Li Z, Lu E, Sheng Q, Zhao Y (2021). Berberine exerts neuroprotective activities against cerebral ischemia/reperfusion injury through up-regulating PPAR-γ to suppress NF-κB-mediated pyroptosis. Brain Research Bulletin.

[262] Zhao Y, Lin X, Zeng W, Qin X, Miao B, Gao S (2023). Berberine inhibits the progression of renal cell carcinoma cells by regulating reactive oxygen species generation and inducing DNA damage. Mol Biol Rep.

[263] Zhao Y, Roy S, Wang C, Goel A (2022). A combined treatment with berberine and andrographis exhibits enhanced anti-cancer activity through suppression of DNA replication in colorectal cancer. Pharmaceuticals.

[264] Zheng C, Wang Y, Xu Y, Zhou L, Hassan S, Xu G (2021). Berberine inhibits dendritic cells differentiation in DSS-induced colitis by promoting Bacteroides fragilis. International Immunopharmacology.

[265] Zheng F, Wu J, Tang Q, Xiao Q, Wu W, Hann SS (2018). The enhancement of combination of berberine and metformin in inhibition of DNMT1 gene expression through interplay of SP1 and PDPK1. J Cell Mol Med.

[266] Zhong L, Lin Y, Gong S, Wu X, Liu Y, Chen J (2023). Oxyberberrubine, a novel liver microsomes-mediated secondary metabolite of berberine, alleviates hyperuricemic nephropathy in mice. Phytomedicine.

[267] Zhong X, Deng H, Long M, Yin H, Zhong Q, Zheng S (2023). Discovery of berberine analogs as potent and highly selective p300/CBP HAT inhibitors. Bioorg Chem.

[268] Zhou R, Huang Y, Tian C, Yang Y, Zhang Z, He K (2023). Coptis chinensis and Berberine Ameliorate Chronic Ulcerative Colitis: An Integrated Microbiome-Metabolomics Study. Am J Chin Med.

[269] Zhu J, Lu H, Guo C, Fang W, Zhao H, Zhou J (2018). Berberine attenuates ischemia–reperfusion injury through inhibiting HMGB1 release and NF-κB nuclear translocation. Acta Pharmacol Sin.

[270] Zhu L, Gu P, Shen H (2019). Protective effects of berberine hydrochloride on DSS-induced ulcerative colitis in rats. International Immunopharmacology.

[271] Zhu N, Li J, Li Y, Zhang Y, Du Q, Hao P (2020). Berberine protects against simulated ischemia/reperfusion injury-induced H9C2 cardiomyocytes apoptosis In vitro and myocardial ischemia/reperfusion-induced apoptosis In vivo by regulating the mitophagy-mediated HIF-1α/BNIP3 pathway. Front Pharmacol.

[272] Zhu X, Bian H, Wang L, Sun X, Xu X, Yan H (2019). Berberine attenuates nonalcoholic hepatic steatosis through the AMPK-SREBP-1c-SCD1 pathway. Free Radical Biology and Medicine.

[273] Zhu Y, Li J, Zhang P, Peng B, Li C, Ming Y (2023). Berberine protects hepatocyte from hypoxia/reoxygenation-induced injury through inhibiting circDNTTIP2. PeerJ.

[274] Zhuang J, Zhang H, Wu J, Hu D, Meng T, Xue J (2024). Redox‐Responsive AIEgen Diselenide‐Covalent Organic Framework Composites Targeting Hepatic Macrophages for Treatment of Drug‐induced Liver Injury. Small.

[275] Zhuang W, Huang Z, Yu L, Yu M, He H, Deng Y (2025). Berberine enhances autophagic flux to alleviate ischemic neuronal injury by facilitating N-ethylmaleimide-sensitive factor-mediated fusion of autophagosomes with lysosomes. Biochemical Pharmacology.

